# Phospholipase D: emerging therapeutic targets in signaling, metabolism, and immune-oncology

**DOI:** 10.1186/s12964-025-02596-z

**Published:** 2025-12-20

**Authors:** Lin Tang, Yunxiao Ge, Yuanying Li, Xiaobing Chen, Kangdong Liu, Zigang Dong, Hui Liu

**Affiliations:** 1 Department of Pathophysiology, School of Basic Medical Sciences, College of Medicine, Zhengzhou University, Zhengzhou, Henan China; 2 Department of Physiology and Pharmacology, Karolinska Institutet, Stockholm, Sweden; 3 Department of Oncology, The Affiliated Cancer Hospital of Zhengzhou University & Henan Cancer Hospital, Zhengzhou, China

**Keywords:** Phospholipase D, Signal transduction, Metabolism, Immunity, Tumor microenvironment, Human diseases

## Abstract

The phospholipase D (PLD) family, comprising six evolutionarily conserved isoforms (PLD1-6), serves as master regulators of lipid signaling, membrane dynamics, and cellular communication. Functional divergence driven by structural heterogeneity enables PLD-mediated control of signal transduction, metabolic homeostasis, and immune responses. Activated by stimuli like growth factors and hormones, PLD governs core signaling networks, including Wnt/β-catenin, protein kinase C (PKC), and mammalian target of rapamycin (mTOR) pathways while modulating glucose uptake and lipid metabolism. PLD isoforms coordinate adaptive and innate immunity through T/B cell activation, macrophage polarization, and cytokine regulation. Dysregulated PLD activity promotes metabolic syndrome, autoimmune diseases, and remodeling of the tumor immune microenvironment, positioning PLD as a therapeutic target. This review integrates isoform-specific mechanisms in signaling, metabolism, immunity and tumor microenvironment, and underscores the critical need for isoform-specific inhibitors to dissect pathological mechanisms and advance disease understanding. By deconvoluting PLD’s pleiotropic roles across signaling axis, lipid-glucose crosstalk, and immune circuitry, this work delineates a roadmap for developing targeted combinatorial therapies that exploit PLD’s spatial-temporal regulation of cellular homeostasis.

**Graphical Abstract:**

The phospholipase D (PLD) enzyme family serves as a central regulatory node governing interconnected biological processes, including cellular signal transduction, metabolic reprogramming, and immunomodulatory networks, particularly within tumor-immune microenvironment dynamics. A comprehensive understanding of PLD's molecular underpinnings in these cascades holds significant translational potential for developing targeted therapies against cancer, autoimmune disorders, and metabolic diseases.

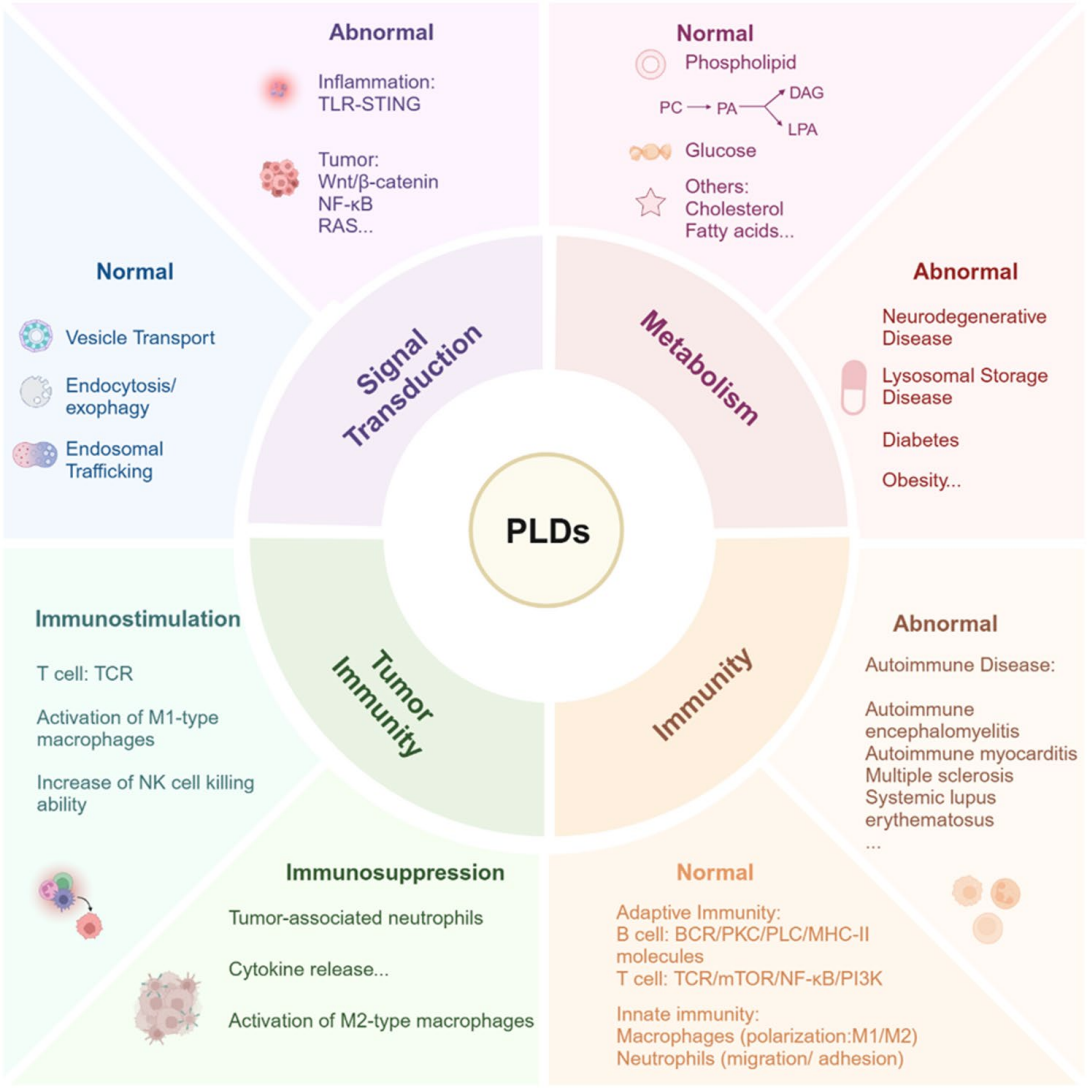

**Facts**

- ▸ Canonical PLD1/2 generate PA (via phospholipase activity) to regulate lipid-glucose metabolism, while non-canonical PLD3/4 use acyltransferase/exonuclease activities to control lysosomal lipid metabolism and immune balance.
- ▸ PLDs critically regulate immune cell functionality (T cells, B cells, neutrophils, macrophages) and associated signaling pathways, offering therapeutic potential for immune diseases.
- ▸ PLD1/2 exerts multidimensional roles within the tumor immune microenvironment (TIME) via lipid signaling, influencing tumor progression, immune evasion, and therapy resistance. PLD3/4 are specifically expressed in tumor-infiltrating immune cells.
- ▸ Future inhibitor development needs to move beyond simple enzyme activity inhibition towards function-specific regulation, considering specific functions and organ/tissue distribution in diseases.

**Open questions**

- ▸ How do non-canonical PLD5/6, with unknown mechanistic roles contribute to diseases, and what strategies define their mechanisms?
- ▸ What is the therapeutic potential of targeting PLD3/4 in tumor-infiltrating immune cells, and how do their exonuclease/lipid metabolic activities shape the TIME?
- ▸ How can inhibitors overcome isoform specificity, functional duality (tumor vs. immune cells), and non-classical PLD challenges (e.g., tissue paradox, dual activities) for cell-specific therapy?

## Introduction

Phospholipase D (PLD), a conserved phospholipase superfamily spanning prokaryotes to eukaryotes, is mainly involved in hydrolyzing phosphatidylcholine to generate phosphatidic acid (PA), a key lipid messenger [1]. Mammals express six isoforms (PLD1–6) classified into canonical PLDs (PLD1/2/6), which are catalytically active enzymes regulating membrane trafficking and signaling; and non-canonical PLDs (PLD3/4/5), which are enzymatically divergent members involved in nucleic acid degradation (PLD3/4) with unresolved functions (PLD5) [2–4]. All PLDs share a catalytic HKD (HxKxxxxD: H: histidine; K: lysine; D: aspartic acid; x: any amino acid) motif. However, canonical isoforms possess additional regulatory domains essential for subcellular targeting and activation.

PLDs are implicated in a broad spectrum of cellular physiological activities, including regulating cytoskeleton dynamics, membrane transport, vesicle trafficking, cell proliferation and migration, inflammatory responses, and organelle functions. Dysregulation of PLDs can lead to abnormal signaling pathways, altered metabolic processes, and impaired immune responses. Several reviews have focused on canonical PLDs’ roles in lipid signaling and disease, including cancer, metabolic disorders, and autoimmune diseases [5–7]. However, there remains a significant knowledge gap regarding the integration mechanism and pathological physiological significance of the entire PLD superfamily, especially for the dual enzymatic functions, phospholipase [4, 8] and nuclease activities [9], of non-classical PLDs and their interactions with immune signaling pathways. This review aims to delineate a coordinated regulatory network encompassing all six PLDs, elucidating interconnected roles in driving cancer progression regarding metabolic dysregulation and immune response, with a particular focus on the emerging functions of non-classical PLDs. Critically, the paradoxical behavior of PLDs across disease contexts creates significant therapeutic hurdles. This review comprehensively analyzes the various regulatory characteristics of PLDs to search for new combination therapy strategies, including the development of subtype-specific inhibitors combined with specific tissue and organ delivery systems. This work not only fills the key knowledge gap but also opens a new path for disease intervention by utilizing the plasticity of the PLDs.

## PLDs activity, cellular localization, and biological function

### PLD1 and PLD2

**Fig. 1:**
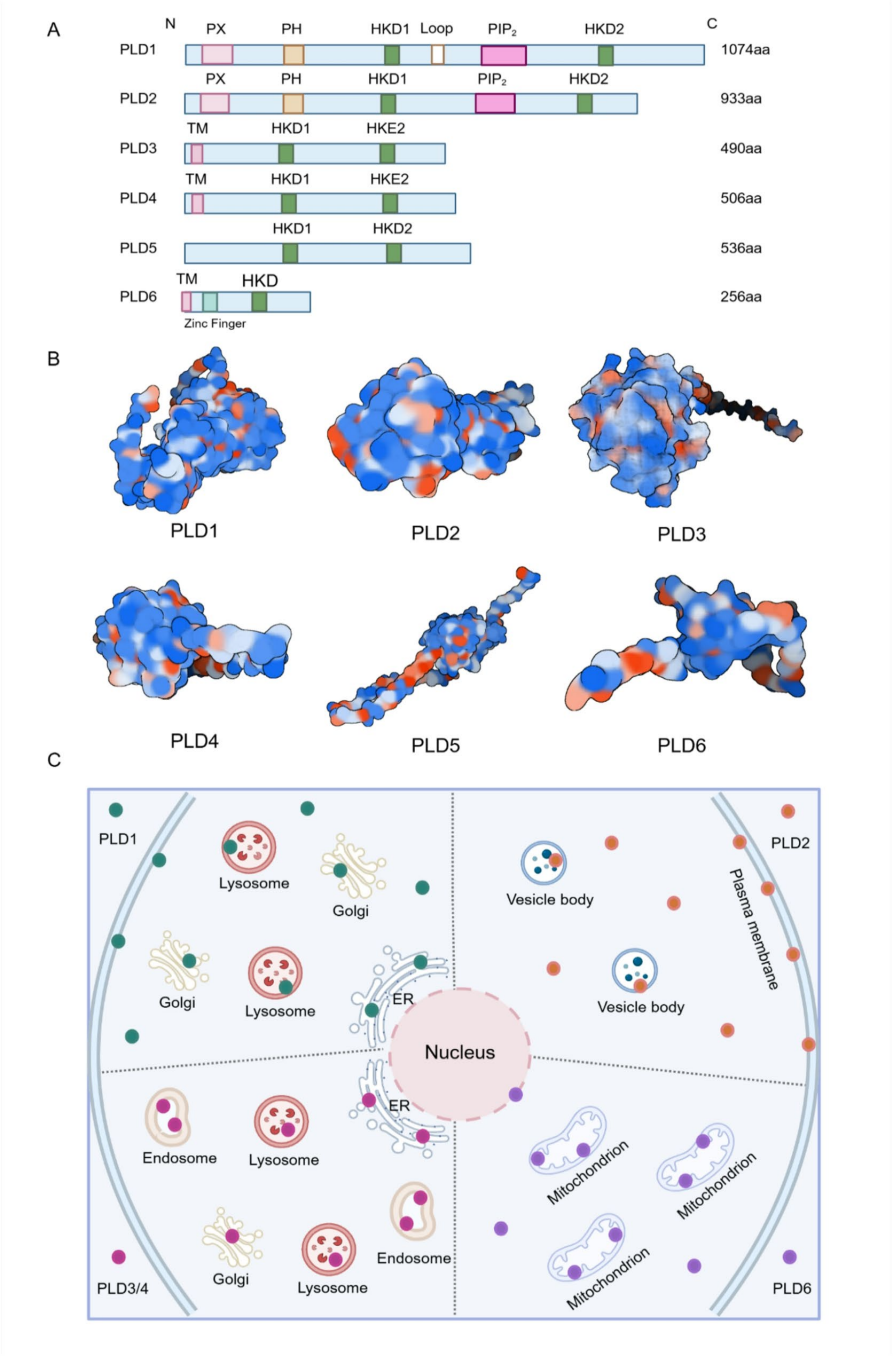
The protein structure and subcellular localization of PLDs. **A**. The amino acid sequence of PLDs: HKD: specific catalytic motifs of PLDs; Loop: region exclusively present in PLD1; PX: phox consensus sequence, present in PLD1, PLD2; PH: pleckstrin homology, present in PLD1, PLD2; PIP_2_: acidic PI [4, 5]P2 binding motif, present in PLD1, PLD2; TM: trans-membrane domain, occurs in non-classical PLDs **B**. Predicted 3D structure of PLDs generated by AlphaFold: PLD1: Q13393; PLD2: O14939; PLD3: Q8IV08; PLD4: Q96BZ4; PLD5: Q8N7P1; PLD6: Q8N2A8 **C**. The subcellular localization of PLDs: PLD1 is predominantly localized to perinuclear and intracellular membranes associated with the Golgi apparatus, endoplasmic reticulum, and lysosomes. PLD2 is primarily localized to the plasma membrane, although it can also be detected in the cytoplasm and vesicular structures. PLD3 and PLD4 are localized to the endoplasmic reticulum, Golgi apparatus, lysosomes, and endosomes. PLD6 is specifically localized to the mitochondrial membrane. ER: endoplasmic reticulum

PLD1 and PLD2, as classical phospholipases, hydrolyze phosphatidylcholine to produce phosphatidic acid and free choline. Structurally, in addition to the two catalytic HKD motifs, PLD1 and PLD2 contain a phox consensus sequence (PX) domain facilitating membrane association by binding to phosphoinositides, a pleckstrin homology (PH) domain (play important role in sensing and responding to cellular signals via lipid interactions), and a phosphatidylinositol 4,5-bisphosphate (PIP2) binding domain, which is crucial for their localization and activation at the plasma membrane [10]. Based on structural studies, HKD motifs are required for catalytic activity, and they dimerize forming active centers [11, 12]. Loop differences between the two motifs distinguish PLD1 from PLD2 **(**Fig. 1A-B**)** [13, 14]. PX and PH domains play important roles in regulating phospholipase D activity, which non-canonical PLDs lack these domains. In addition, the PH domain is also thought to play a role in protein localization, as its deletion or point mutation can inhibit the membrane targeting of PLD1 and alter its cellular sublocalization [15]. Human PLDs must rely on the membrane-lipid phosphatidylinositol- [4, 5]-bisphosphate (PI [4, 5]P_2_) as a cofactor, the PIP2 binding site facilitating its catalytic activity [16].

PLD1 differs from PLD2 in cellular localization. PLD1 is localized in perinuclear, intracellular membranes of Golgi apparatus, endoplasmic reticulum, and lysosomes **(**Fig. 1C**)** [17–19]. Upon stimulation of the cells by some exogenous substances (such as IgE secretion by mast cells (RBL-2H3) [20], phorbol esters stimulation of fibroblasts and COS-7 cells [21]), leading to the translocation of proteins to the plasma membrane. PLD2 predominantly resides on plasma membranes under normal conditions [22], but also be found in the cytoplasm, vesicular bodies **(**Fig. 1C**)** [23, 24], and associated with β-actin. Agonist stimulation results in co-localization of PLD2 receptors to the plasma membrane and endocytic vesicles. PLD2 has been reported to be involved in receptor endocytosis and recycling [25]. Thus, the cellular localization of PLD1 and PLD2 is closely related to their functional roles, including cytoskeletal reorganization membrane transport, and vesicle trafficking. Agonists of PLD1 and PLD2 have been reported in detail in the previous review article [26]. They require the regulation of agonists to exert enzymatic activity to produce PA and participate in cell signaling transduction.

**Fig. 2:**
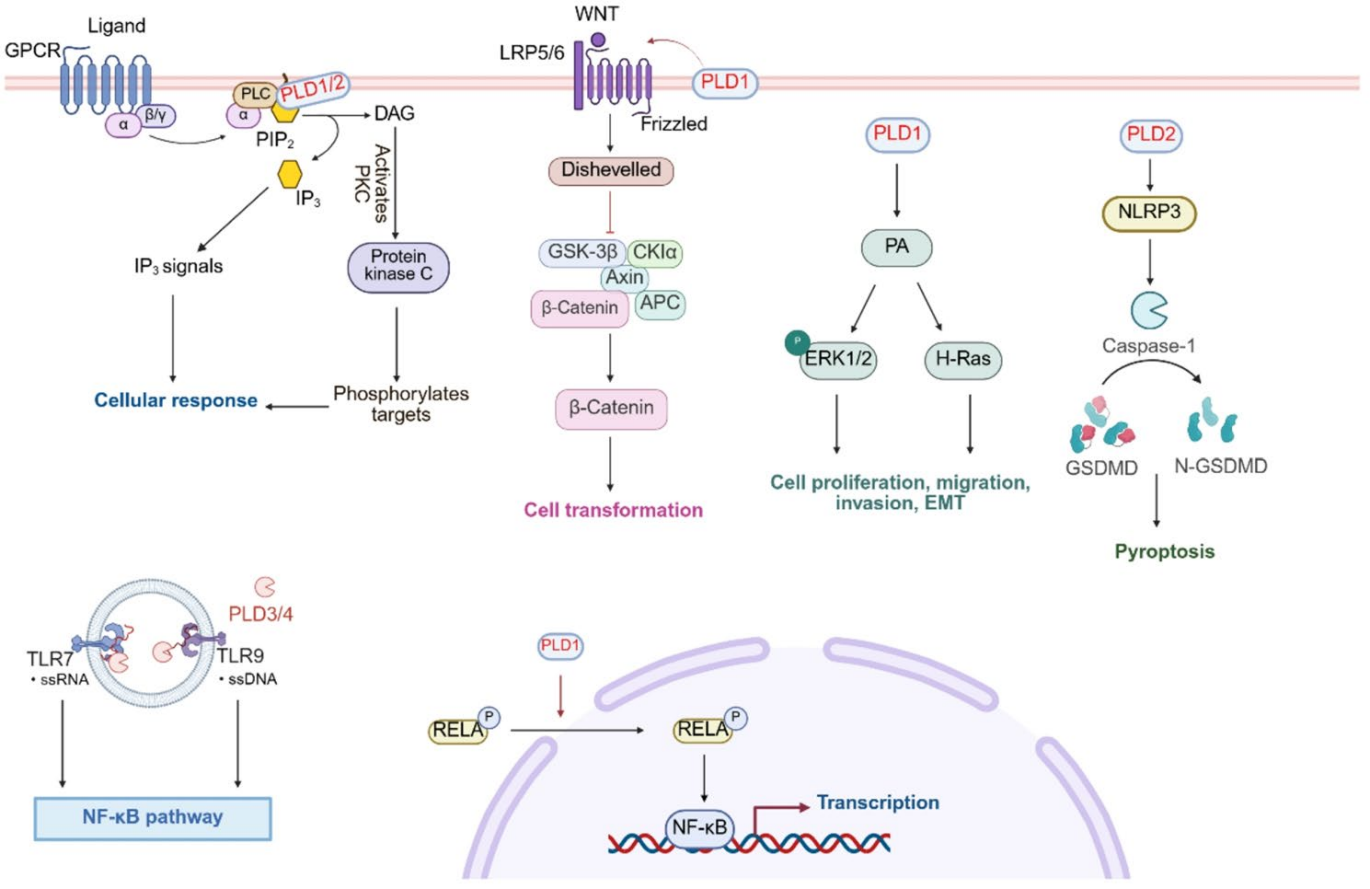
PLDs-mediated integration of signaling networks. PLD1/2 are involved in the metabolism of PIP2 on the cell membrane and activate the downstream signal transduction IP3 and DAG. IP3 regulates the release of calcium ions and triggers the intracellular signal cascade. DAG participates in the activation of PKC and regulates the physiological response of cells. PLD1 can enhance Wnt/beta-catenin signaling associated with cell transformation. PLD1 activates NF-κB signaling via p65/RELA phosphorylation-driven nuclear import. The PA derived from PLD1 promotes tumor proliferation, migration, invasion, and EMT by activating H-Ras and ERK1/2. PLD2 regulates myocardial inflammation and pyroptosis through the NLRP3/caspase 1/GSDMD pathway. PLD3 and PLD4 regulate lysosomal homeostasis through exonuclease activity and participate in Toll-like signaling pathway, assisting TLR7 to recognize RNA (PLD3) and negatively regulating TLR9 to recognize DNA (PLD4)

PA produced by PLD1/2 can mediate a variety of cellular functions, such as proliferation, vesicular trafficking, and cytoskeleton regulation via interacting with signaling molecules [27–29]. The enzymatic activity of PLDs is regulated by several signaling factors, which can be divided into phosphoinositide, small GTPase, protein kinase, structural protein and so on, based on their biological functions. Human PLD1/2 facilitates the metabolism of PIP2 at the cell membrane, catalyzing it to the secondary signaling molecules phosphatidylinositol and diacylglycerol (DAG). By interacting with intracellular stored calcium ions, IP3 releases intracellular calcium ions, thereby triggering an intracellular signaling cascade. DAGs are involved in the activation of protein kinase C (PKC) and then regulate the physiological response in cells (Fig. 2).

### PLD3-PLD6

Compared with other members of PLD, although PLD3 and PLD4 show only 28% identity between their amino acid sequences, they show the greatest homology between HKD1 and HKE2 domains [30]. PLD3 and PLD4, respectively, contain 490 and 506 amino acids (AA). They lack PX and PH domains, and aspartic acid is replaced by glutamic acid in the second HKD motif. In recent years, PLD3 and PLD4 have been found to have exonuclease activities that are highly similar to those of splenic exonuclease or phosphodiesterase enzymes described previously [9, 31, 32]. They are type II transmembrane proteins that each contain a transmembrane (TM) domain (Fig. 1A-B). PLD3 is initially identified as an endoplasmic reticulum (ER)-resident glycosylated protein, with a 38-AA transmembrane amino-terminus and a C-terminal luminal domain of the remaining amino acids. With the depth of researches, PLD3 was found to be localized in lysosomes and involved in lysosomal function [33]. PLD3 is irregularly transported to lysosomes and endosomes through the endoplasmic reticulum and Golgi apparatus (Fig. 1C). The TM fragment of PLD3 binds to the lysosomal membrane, and the lumen contains the catalytic active domain of HKDs and the glycosylation site. The full-length PLD3 is hydrolyzed by lysosomal proteases to produce a soluble luminal domain and a TM fragment [34].

The cellular localization of PLD4 is highly similar to that of PLD3, both being lysosomal proteins (Fig. 1C). They exert exonuclease activity in lysosomes to regulate the recognition of nucleic acid-sensing Toll-like receptors (TLRs). In the past, PLD3 and PLD4 were thought to be PLD members without phospholipase activity. However, recent studies have shown that they catalyze the synthesis of S, S- Bis (monoacylglycero) phosphate (BMP), a key lysosomal phospholipid required for lipid degradation, suggesting that they have phospholipase activity [3, 35]. More studies are needed to verify the function and mechanism of PLD3’s and PLD4’s phospholipase activity in biological and pathological processes.

In contrast to other non-classical phospholipase D family members, PLD6, also known as MitoPLD, encodes only a single HKD motif and exerts phospholipase activity in the dimeric form, hydrolyzing cardiolipin (CL) and forming a fusion lipid PA at the mitochondrial outer membrane to mediate mitochondrial fusion [36]. PLD6 also has endonuclease activity, specifically cleaving DNA and RNA substrates. PLD6 contains a 256 AA, and 39 AA N-terminal domain that serves as a transmembrane domain, anchoring it to the mitochondrial outer membrane (Fig. 1C) [37]. PLD6 has been reported to be located in cytoplasmic granules and around the cytosol, including the nucleus, possibly in association with its nuclease activity (Fig. 1C) [38]. Few studies have been conducted on PLD5, which has the most similar structural features to PLD3 and PLD4. But its phospholipase activity has not been found so far, probably due to its non-conserved HKD motif (lack of histidine and lysine) (Fig. 1A-B) [39]. More studies are needed to elucidate the activity, cellular localization, and biological function of non-canonical PLDs.

## PLDs in pathological signaling networks

### Cytoskeletal dynamics and oncogenic signaling by canonical PLDs (PLD1/2)

PLD1 and PLD2 play a key role in regulating the actin cytoskeleton by modulating the synthesis of membrane phospholipids. These phospholipids have been demonstrated to form microstructural domains that function as signaling platforms for various cellular processes, including vesicular trafficking and autophagy [40]. Deficiency in PLD results in aberrant megakaryocyte (MK) morphology and impaired podosome formation in response to type I collagen. Specifically, *Pld1* knockout (KO), *Pld2* KO, and double KO mice models exhibit reduced actin filament density and decreased podosome numbers [41]. Phospholipase D signaling network affects tumor cell division, survival, chemotherapy resistance, and angiogenesis by regulating bioactive lipids including lysophosphatidic acid (LPA), phosphatidylinositol 3,4, 5-triphosphate (PIP3), PA and sphingosine 1-phosphate [42]. Emerging as a downstream transcriptional effector of the β-catenin/T cell factor (TCF) complex, PLD1 exerts oncogenic effects through bidirectional potentiation of Wnt/β-catenin signaling. This regulatory mechanism not only drives malignant cellular transformation but also facilitates pathological hyperactivation of downstream oncogenic targets, establishing its central role in multiple cancer pathogenesis (Fig. 2) [43]. PLD1 potentiates nuclear factor kappa-B (NF-κB) signaling through a dual mechanism involving phosphorylation at critical residues and nuclear import of the NF-κB subunit (P65), thereby promoting nasopharyngeal carcinoma tumorigenesis [44]. The PA derived from PLD1 promotes the proliferation, migration, invasion, and epithelial-mesenchymal transition (EMT) of cervical cancer through activating H-Rat sarcoma (H-Ras) and extracellular signal-regulated kinase 1/2 (ERK1/2), and the absence of PLD1 inhibits tumor growth, enhances cisplatin sensitivity, and reduces the expression of H-Ras/p-ERK1/2 (Fig. 2) [45]. In addition, PLD1 can induce EMT and activate the integrin family pathways, subsequently activating downstream mitogen-activated protein kinases (MAPK) and protein kinase B (also known as Akt) [46].

PLD2 mediates cytoprotective effects in acute pancreatitis pathogenesis through coordinated activation of the nuclear factor E2-related factor-2 (Nrf2) antioxidant pathway and suppression of NF-κB-mediated inflammatory signaling pathway, with concomitant attenuation of apoptotic cascades, cellular edema, and plasma membrane integrity compromise (Fig. 2) [47]. PLD2 with or without enzymatic activity had different effects on lymphoma cells. Active PLD2 increases the phosphorylation of focal adhesion kinase, activates Akt, and promotes cell invasion in EL4 lymphoma cells, while inactive PLD2 suppresses oncogenic processes encompassing cellular adhesion, migratory potential, tissue infiltration, and neoplastic growth [48]. PLD2 regulates myocardial inflammation and pyroptosis through the NOD-like receptor family pyrin domain containing 3/caspase 1/gasdermin D pathway (Fig. 2) [49]. Direct binding between kinesin family member 5B and PA, produced by PLD2, facilitates the coupling of cargo-carrying membrane type 1 matrix metalloproteinase to microtubules, thereby enhancing their trafficking to the plasma membrane. This process plays a crucial role in the invasion and metastasis of breast cancer cells [50].

### Non-canonical PLDs regulated signaling pathways

The roles of PLD1 and PLD2 in cell signal transduction are dependent on their phospholipase activities. PLD3 and PLD4 are 5’ to 3’ ssDNA/ssRNA exonucleases located in the endolysosomal compartment that exert nuclease activity as luminal soluble proteins. They regulate lysosomal homeostasis through exonuclease activity and participate in Toll-like signaling pathway, assisting Toll-like receptor 7 to recognize RNA (PLD3) and negatively regulating TLR9 to recognize DNA (PLD4) (Fig. 2) [51]. PLD exonuclease has dual roles, acting as both pro-inflammatory and anti-inflammatory factors. If their enzymatic activities are dysregulated, they may cause related inflammatory and immune responses. PLD4, as a potential target in the treatment of systemic lupus erythematosus (SLE), affects the function of T cells or B cells in SLE by regulating TLR7 or TLR9 signaling [52]. Furthermore, PLD3 is involved in the trafficking of ER-associated vesicles in myoblasts and is essential for myotube formation. However, its mechanism of action has not been fully elucidated [53]. PLD6 is responsible for the hydrolysis of mitochondrial outer membrane localized cardiolipin to phosphatidic acid and participates in mitochondrial lipid signal transduction [54] and homeostasis regulation [55]. Diseases and signaling pathways related to PLD5 remain deserted, which need to be further elucidated.

## PLD-driven coordination of glycolipid metabolism

### PLDs are involved in phospholipid metabolism

Lipid metabolism refers to the synthesis, decomposition, and transformation of lipid molecules, such as fatty acids, triglycerides, cholesterol, etc. The main roles of lipids are to provide energy, constitute cell membranes, serve as signaling molecules, and participate in hormone synthesis. Phospholipid metabolism is one of the core processes of lipid metabolism, which can be divided into glycerophospholipids and sphingomyelins according to their structural differences. Glycerophospholipids, the most abundant phospholipids in the human body, composition and surfactant production, and facilitate membrane-mediated protein recognition and signaling cascades. The basic structure of glycerophospholipids is phosphatidic acid and a substituent group X (X: choline, ethanolamine, serine, glycerol, inositol) attached to phosphate. They are classified according to group type into a number of classes, including phosphatidylcholine (PC), phosphatidylethanolamine (PE), phosphatidyglycerol (PG), phosphatidylinositol (PI), phosphatidylserine (PS), and CL. Sphingolipid, characterized by the absence of glycerol and the presence of sphingosine, can be divided into sphingomyelin and glycosphingolipid according to the substituted group X (X: phosphorylcholine, glycosyl).

**Fig. 3:**
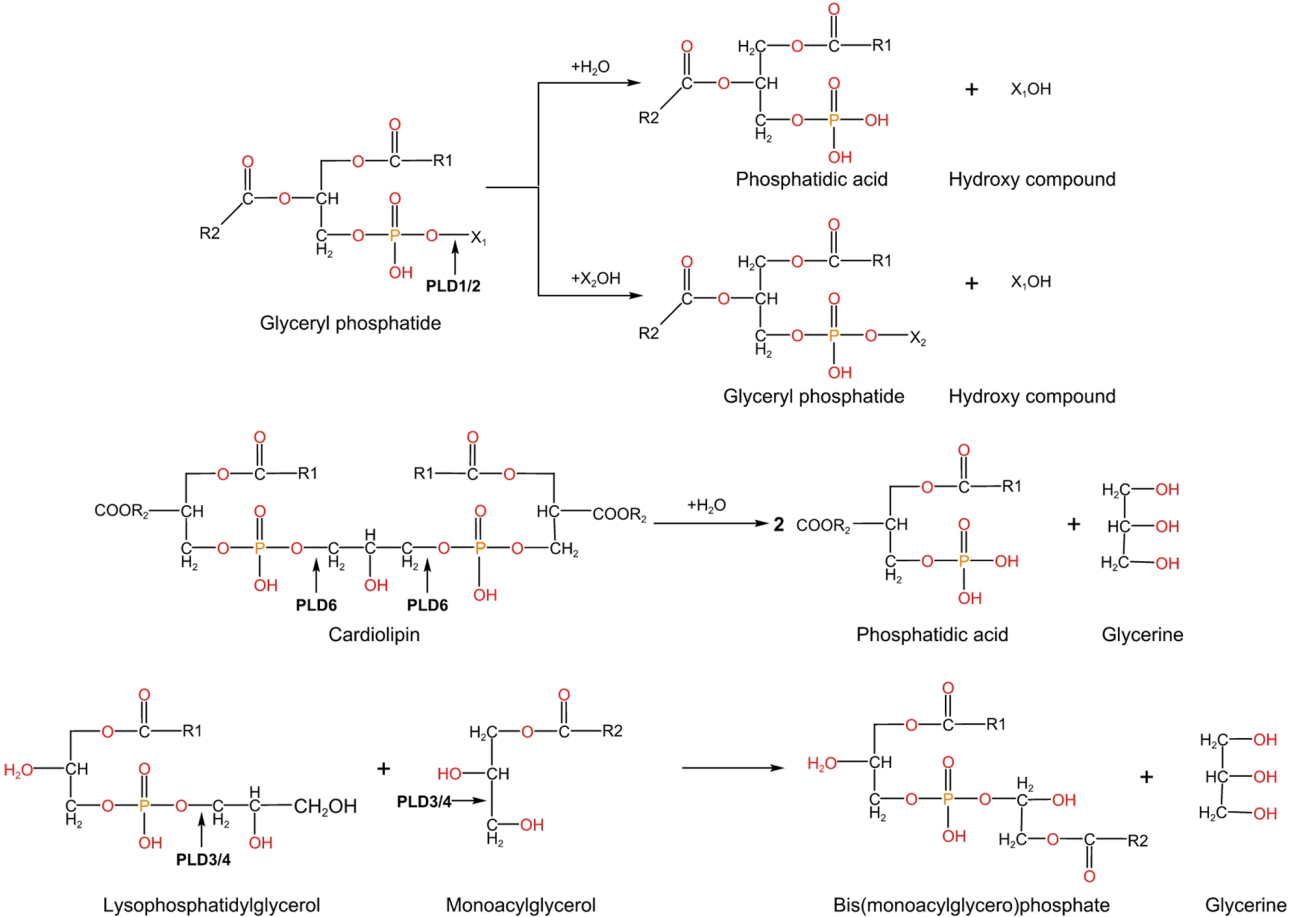
Catalytic reactions of PLDs. PLD superfamily exhibits distinct substrate preferences and catalytic functions across its isoforms, the canonical PLD1/2 is implicated in the hydrolysis or transphosphatidylation of phospholipid, PLD6 is associated with the hydrolysis of cardiolipin, and PLD3/4 mediates bis (monoacylglycerol) phosphate (BMP) synthesis through acyltransferase-independent remodeling of phospholipid acyl chains

PLD1 and PLD2 are mainly involved in phospholipid catabolism through its activity of hydrolyzing PE and trans-phosphatidyl activity (Fig. 3). PA produced from PLDs can be further converted to DAG and LPA by phosphatidic acid phosphohydrolases (PAP) family and phospholipase, respectively [56, 57]. PAP2b co-localizes to PLD2-rich membrane domains, and this association contributes to the rapid conversion of PLD-produced PA to DAGs involved in the activation of the classical PKC family [58]. The interconversion between DAG and PA is dynamically regulated through diacylglycerol kinases (DGK)-mediated phosphorylation reactions. The levels of PA and DAG are tightly controlled by PLDs, PAP, and DGK activities. Phosphatidylinositol is a lipid that serves as the precursor molecule for PI, which can undergo phosphorylation to yield PI [4, 5]P2. Both PLD1 and PLD2 possess a binding domain specific to PI [4, 5]P2 and rely on this lipid for their enzymatic activities. There is bidirectional regulation between PLDs and phospholipid metabolism. PLDs play important roles in phospholipid metabolism, and phospholipids also regulate their PLD enzyme activity by binding to their domains. The phospholipase activity of PLD3 and PLD4, members of the non-classical PLD family, has not been reported in the past [30]. However, the recent study found that they can catalyze the formation of BMP from DAG and monoacylglycerol (Fig. 3) [4]. BMP, also known as lysophosphatidic acid, is a special phospholipid enriched in lysosomal compartments and exosomes. Its unique stereochemical configuration (S, S-BMP) prevents it from being hydrolyzed by the common major phospholipases that hydrolyze PC and PE, thereby regulating lysosomal homeostasis [59]. Lysophosphatidylglycerol is a precursor lipid for BPM synthesis. The transformation of PG into the stereochemical structure of S, S-BPM requires a series of complex and continuous reactions involving a variety of phospholipases, including lysosomal phospholipase A2, transacylase, lysosomal lipase, and PLD3/4. Most phospholipids are widely localized in the cell membrane and organelle membrane. In addition to the special BMP located in lysosomes, there is CL located in mitochondria, also known as diphosphatidylglycerol, which is a mitochondrial-specific phospholipid with a unique structure of four acyl chains [60]. CL is synthesized through multiple enzymatic reactions from PA, which is transported from the endoplasmic reticulum (ER) to the inner mitochondrial membrane. An intermediate CDP-diglyceride or PG is catalyzed by CL synthetase resulting in CL production [61]. CL is in turn hydrolyzed back to PA by PLD6 localized within mitochondria (Fig. 3) [62]. PLD6 affects mitochondrial structure and function, including mitochondrial respiration, ATP production and apoptosis, by regulating CL and PA metabolism and remodeling in mitochondria.

**Fig. 4:**
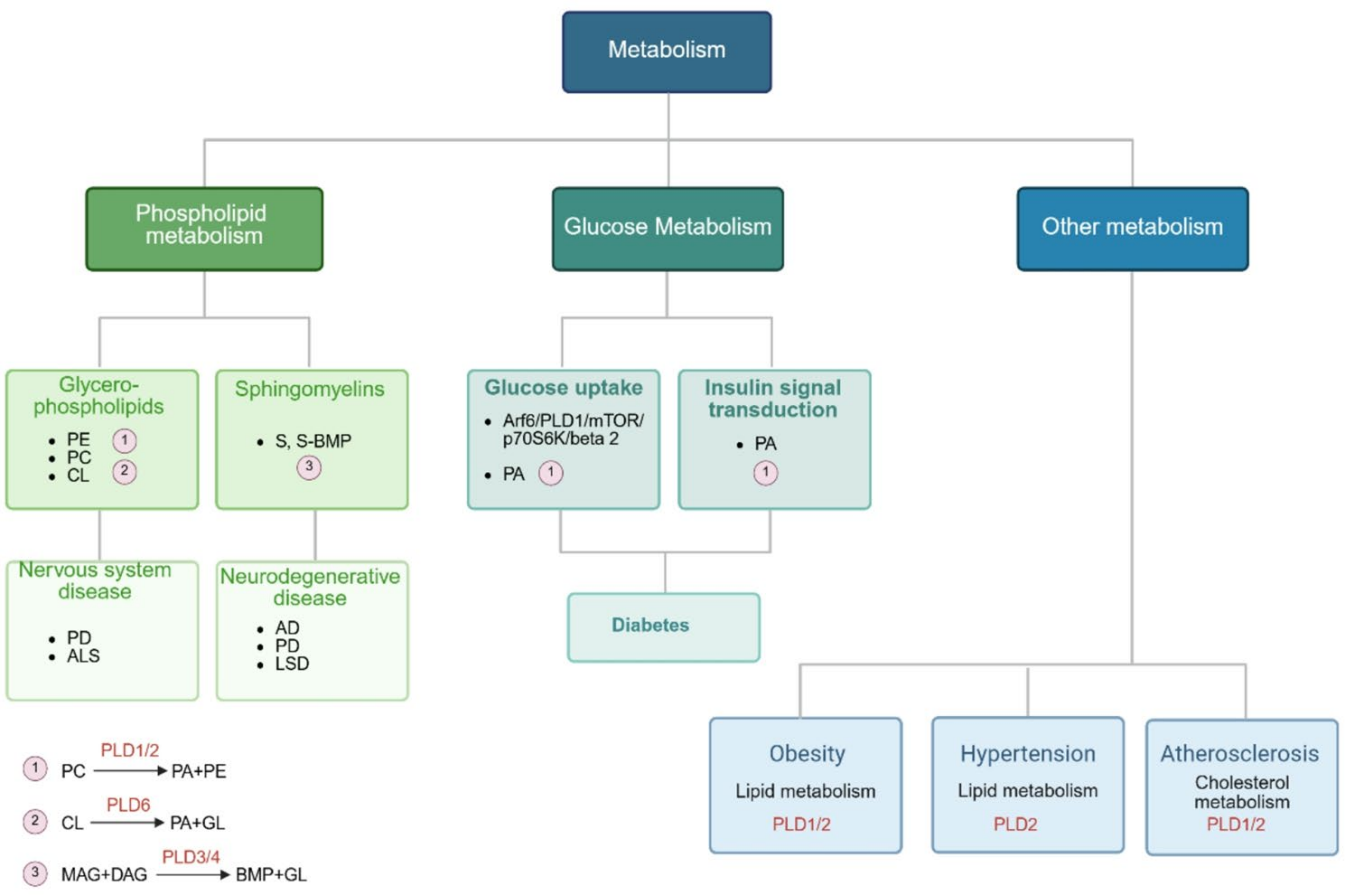
PLDs in regulating metabolic processes. PLDs function as central metabolic orchestrators, intricately regulating phospholipid homeostasis through PA-mediated signaling cascades. PLDs also contribute significantly to glucose metabolism, influencing how cells absorb and utilize glucose as an energy source. Furthermore, its involvement in cholesterol metabolism highlights its importance in regulating lipid levels and ensuring proper cellular function. PLDs disorders can cause metabolic disorders, thereby promoting the occurrence and development of related diseases (such as neurodegenerative diseases, diabetes, hypertension, etc.)

Emerging evidence implicates phospholipid signaling in synaptic transmission and neuropathology. Mechanistically, PA biosynthesis via PLD activation correlates with vesicular trafficking, as demonstrated in leukocyte secretion models [63]. Within the central nervous system, PLD1 predominantly localizes to neuronal populations while PLD2 shows astrocytic expression patterns. Neuronal PLD1 facilitates vesicle-plasma membrane fusion through PA-enriched microdomain formation that coordinates vesicular docking machinery [64]. These results suggest that dysregulation of PLD can cause changes in the content and ultrastructure of PA, which are key factors leading to nervous system diseases. Abnormal levels of PA are associated with cognitive decline [65], PA may affect the function of dopaminergic neurons by regulating cell survival and apoptosis pathways in Parkinson’s disease (PD) [66], and modulation of PA may influence neuronal survival and recovery processes after ischemia **(**Fig. 4**)** [67].

BMPs are significantly decreased in cells or mouse tissues depleted of PLD3 and PLD4 [4]. BMP-mediated lipid degradation is essential for cellular function, and the decrease of BMP content can lead to the occurrence of a variety of diseases, especially neurodegenerative diseases, such as Alzheimer’s Disease (AD) and PD, and lysosomal storage diseases. Impaired BMP signaling disrupts metabolic clearance of sialylated glycosphingolipids predominantly localized to neural tissues, with consequent pathological buildup precipitating neurodegeneration through neurotoxic mechanisms [68, 69]. The abundance of BMPs and other lipids such as cholesterol is closely correlated within the cell, and an alarming accumulation of BMPs has been reported in the literature in Niemann-Pick disease, a lysosomal cholesterol storage disorder [70, 71]. In addition to promoting cholesterol transfer, excess BMP accumulation may also trigger extracellular vesicle shedding or activation to release cholesterol loading fluid in Niemann-Pick disease [72, 73]. The over-activation or inactivation of PLD3 and PLD4 can change the content of BMP, which leads to the occurrence of lipid-related diseases, especially neurodegenerative diseases. PLD3 was previously reported to be an independent risk factor for Alzheimer’s Disease, and coding abnormalities of this gene increase the risk of AD [3]. The specific molecular mechanism of PLD3 affecting the occurrence and development of AD has not been fully elucidated, and it may be related to the regulation of lipid metabolism by its phospholipase activity **(**Fig. 4**)**.

CL synthesis and remodeling are significantly affected by a range of phospholipases. In both AD patients and AD mice models [74, 75], CL content is decreased, and low CL content correlates with impaired mitochondrial synaptic function and oxidative stress both in vitro and in vivo. Altered CL levels have also been implicated in amyotrophic lateral sclerosis (ALS), a fatal neurological disease, reduced CL levels in the spinal cord as well as other lipidomic changes observed in ALS mouse models [76]. CL abnormalities affect a variety of nervous system diseases, but the contribution of CL abnormalities to neurodegeneration is not fully understood, and the role of PLD6 as a CL hydrolase needs to be further explored. PLDs dysregulation can significantly affect phospholipid metabolism. PLD1/2 regulates membrane phosphatidic acid, PLD3/4 affects lysosomal BMP content, and PLD6 regulates CL, leading to neurodegenerative diseases. Enhancing our understanding of the PLD family’s role in phospholipid metabolism is crucial for comprehending the molecular mechanisms and developing treatment strategies for neurodegenerative diseases **(**Fig. 4**)**.

### PLDs participate in glucose metabolism

PLDs also play key roles in glucose metabolism. Insulin can activate PLDs by stimulating adipocyte surface receptors to promote the translocation of ADP-ribosylation factor protein to the cell membrane [77]. Treating pancreatic β-cells with glucose can lead to activate PLD1 and increase protein expression by Arf6/PLD1/mTOR/p70S6K/beta 2 pathway [78]. The activation of PLDs can enhance glucose uptake in response to insulin, insulin-stimulated PLDs activation in primary hepatocytes is linked to increased glucose uptake and glycogen synthesis [79], as PA has been shown to stimulate translocation of glucose transporter type 4 to the plasma membrane [80]. Conversely, overexpression of either PLD1 or PLD2 inhibited insulin signaling in primary mouse hepatocytes and was accompanied by an increase in the cellular content of total PA [81], highlighting the enzyme’s dual role in glucose homeostasis. PLD isoforms demonstrate pathophysiological significance in diabetic progression, particularly through modulation of insulin signaling pathways. In pancreatic β-cell models, epidermal growth factor receptor activation was found to potentiate both PLD enzymatic function and insulin exocytosis, whereas PLD2 genetic ablation attenuated this secretory response [82]. These mechanistic insights establish PLD2 as a critical mediator of growth factor-regulated insulin dynamics, highlighting its therapeutic candidacy for glycemic control strategies. In type 2 diabetes, PLD-derived PA orchestrates molecular crosstalk with insulin receptor substrate (IRS) proteins, dysregulating phosphorylation kinetics and signal propagation to drive systemic insulin desensitization and diminished cellular responsiveness [83]. However, a fundamental paradox emerges, while PLD-generated PA disrupts peripheral insulin signaling (pathological lipotoxicity), it simultaneously potentiates insulin secretion in β-cells via growth factor-dependent exocytosis (physiological signaling). This dichotomy underscores the dual regulatory role of PA concentration thresholds and tissue microenvironments in governing PLD2 function. Consequently, therapeutic targeting demands precise spatiotemporal balance, inhibiting peripheral PLD2 may ameliorate insulin resistance but risks impairing β-cell secretory capacity. Thus, unlocking PLD2’s therapeutic potential hinges not only on deciphering its phospholipid-protein interactions but also on resolving this tissue-specific functional conflict. Understanding the precise mechanisms by which PLD influences glucose metabolism is crucial for developing therapies for type 2 diabetes and related metabolic disorders **(**Fig. 4**)**.

PLDs are also involved in the regulation of other metabolic diseases, such as obesity, hypertension, and atherosclerosis. Obesity, a condition caused by the accumulation of excessive body fat, especially triglycerides, refers to a certain degree of significant weight and fat layer thickness. Hypertension, referred to as the silent killer, is a significant risk factor for various cardiovascular diseases. *Pld1* and *Pld2* deletion caused increased overweight and development of diabetes in mice [84]. PLD-specific PA inhibited peroxisome proliferator-activated receptor γ activity [85], a receptor associated with increased food intake during nervous system activation. Based on the animal experiment, it was found that *Pld2* knockout was linked to a marked elevation in blood pressure, body weight, and hyperlipidemia compared with the control group **(**Fig. 4**)** [86]. Atherosclerosis, a disorder caused by abnormal deposits of lipids in the blood vessels, which can be caused by diabetes, obesity, and hypertension. Atherosclerosis is a complex immune-mediated metabolic-inflammatory disorder, pathologically defined by subendothelial accumulation of cholesteryl esters that drives pathological vascular remodeling. PLDs regulate the metabolism of high-density lipoprotein and may affect the reverse transport of cholesterol, that is, cholesterol is transported from peripheral tissues to the liver for excretion **(**Fig. 4**)** [87]. Metabolic-related diseases are basically involved in lipid metabolism (including phosphatidic acid, cholesterol, fatty acid, and triglyceride) and glucose metabolism, etc. The role of PLDs in metabolic diseases has not been fully elucidated. Further exploration of the different roles of the PLD family in metabolic pathologies may strategically inform the development of isoform-selective therapeutic modalities for precision management of these conditions.

## PLDs in immunity: critical regulators of immune cell coordination and autoimmune disease pathogenesis

### Modulation of adaptive and innate immune responses by PLDs

Immunity, a physiological function of humanity, can function through precise self-nonself discrimination to neutralize exogenous antigens and eliminate autologous aberrant cells, including those undergoing malignant transformation, thereby preserving the homeostatic equilibrium of the organism. Immunity is classified as innate and adaptive based on specific and nonspecific responses. PLD family exhibit ubiquitous expression across immune cell lineages, spanning myeloid-derived phagocytes to lymphoid lineage effectors, where they orchestrate functionally divergent mechanisms in both evolutionarily conserved innate defenses and antigen-specific adaptive responses.

#### PLDs regulate adaptive immune cells

**Fig. 5:**
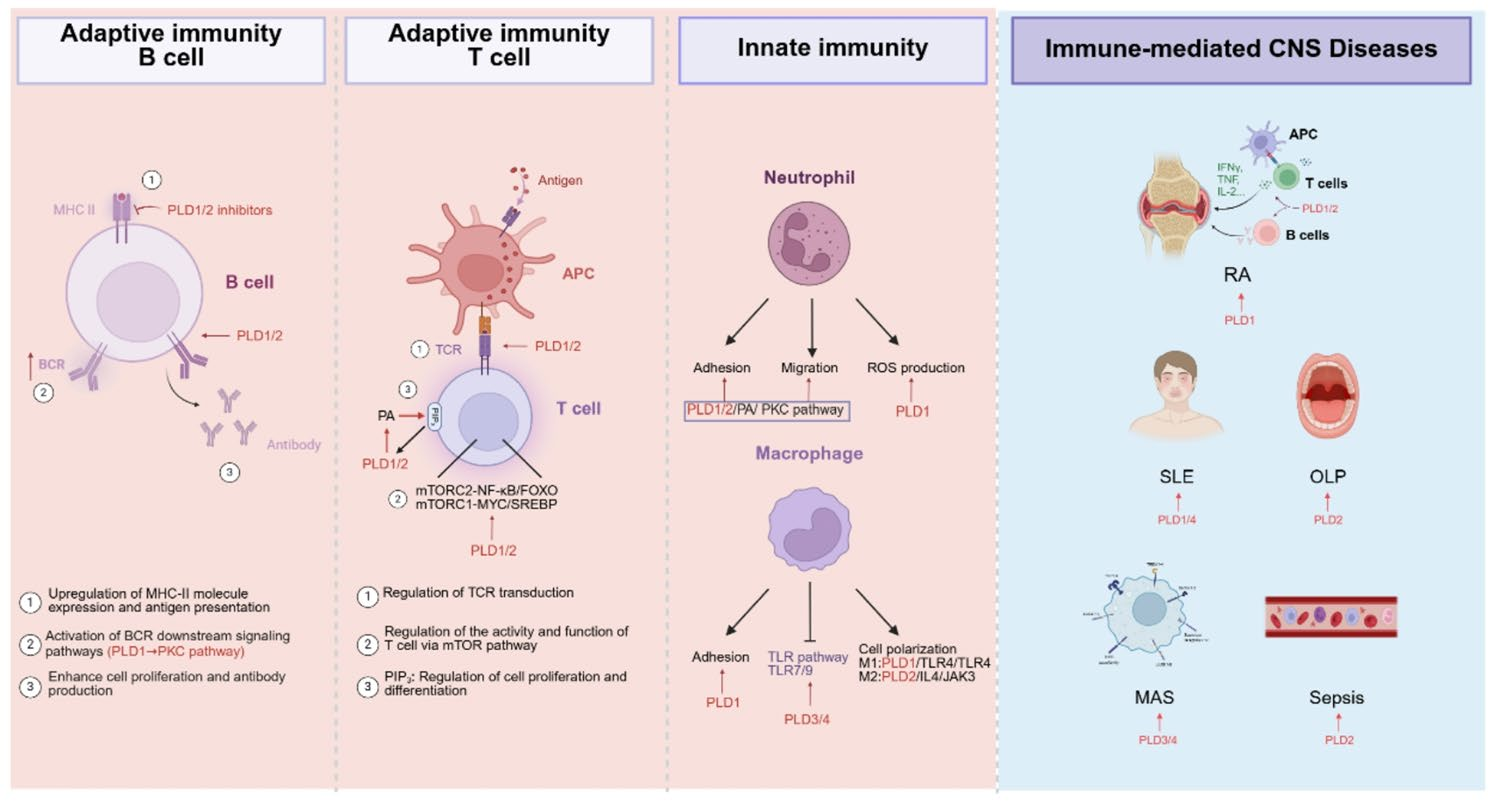
PLDs participate in immunity. PLD isoforms, particularly PLD1 and PLD2, serve as critical regulators of both innate and adaptive immunity. In adaptive immunity, PLDs can regulate the expression of MHC-II molecules and antigen presentation, activate the downstream signaling pathway of BCR, promote cell proliferation and antibody production in B cells. PLDs can regulate TCR signaling, regulate cell activity and function through mTOR pathway, and regulate cell proliferation and differentiation by binding to PIP3. For innate immunity, PLDs are involved in the adhesion, migration, and reactive oxygen species production of neutrophils. PLDs also play crucial regulatory roles in the adhesion, polarization, and inflammatory response of macrophages. Disruptions in PLDs function can lead to immune system disorders and are linked to various autoimmune diseases

PLDs can hydrolyze PIP_2_ to produce phosphatidylinositol-4-phosphate and DAG, and then regulate the activation, proliferation, and differentiation of B cells. It has been found that PLDs play important roles in B-cell receptor-mediated signaling pathways, such as by activating PKC and PLC pathways [88]. Stimulation of B cell surface receptors (BCR) can trigger a cascade of signal transduction events, and among these evolutionarily conserved pathways, protein tyrosine kinase family activation serves as a critical node functionally coupled to PLD-mediated signaling networks [89]. In addition, PLDs activity also regulate B cell immune response. Enhancement of PLDs activity can promote B cell proliferation and antibody production, and increased PLDs activity can promote the expression of MHC-II molecules and antigen presentation, thereby enhancing the activation of T cells [90]. However, the specific mechanism of PLDs activation in B cells has not been fully elucidated and needs further study (Fig. 5).

The associated antigens of T lymphocytes can stimulate and induce the up-regulation of PLDs activity, and inhibition of PLDs signaling blocked the proliferation of effector T cells upon T cell-antigen receptor (TCR) binding, but had no significant effect on the proliferation of regulatory CD4^+^ CD25^+^ T cells [91]. TCR, a key adaptor protein in T cell signaling, functions as a multimeric receptor complex essential for lymphocyte activation cascades. Pathological perturbations in this signaling axis manifest clinically as either immunodeficiency disorders or autoimmune pathologies [92]. mTOR, a key regulatory pathway related to metabolism, can control immune cell biology in a cell type-specific and context-dependent manner [93]. By activating AKT, mTORC2 couples NOTCH inputs to NF-κB/FOXO transduction axis, thereby facilitating thymocyte development [94], while mTORC1 drives T cell activation through MYC/SREBP-mediated metabolic reprogramming [95]. PLD1/2 can induce mTOR expression and activation, implicating a potential role for PLDs in response to signals that require mTOR activation [96, 97]. In addition, activated PLD2 decreased oxidative phosphorylation signaling (a Warburg hallmark) in mitochondria while potentiating glycolysis through mTOR-dependent metabolic reprogramming [98]. These findings significantly suggest that PLDs can regulate the activity and function of T lymphocytes, and its regulatory pathway may be through mTOR signaling pathway **(**Fig. 5**)**.

As a key cofactor of PLDs, PIP2 plays a pivotal role in the activity, membrane localization, and receptor activation of PLD isoforms PLD1 and PLD2. The decrease of PIP2 level in cells could inhibit the activity of PLDs, on the contrary, the PLDs product PA stimulated the activity of phosphatidylinositol 5-kinase (PI5K), phosphorylating PIP2 to produce phosphatidylinositol 3,4, 5-triphosphate. PIP3, as a signal molecule on the cell membrane, participates in the activation and function of a variety of immune cells, T and B cell proliferation, differentiation, and survival [99]. PIP3 produced by PI3K binds to the PH domain in the target protein and activates a signaling cascade. It can promote phosphorylation of the serine kinase PKB, which activates TORC1 for T cell growth, and it can also activate metabolic transitions, such as cytotoxic T-lymphocyte activation [100].

#### PLDs modulate innate immunity

PLDs play important regulatory roles in innate immunity, including monocytes, macrophages, dendritic cells, and neutrophils. In earlier studies, PLDs had regulatory effects on the activation of neutrophil respiratory burst dependent on Ca^+^ activation [101], Trp-Lys-Tyr-Met-Val-D-Met, a peptide that stimulates the hydrolysis of PI in human leukocytes (including monocytes), bound to leukocyte (monocytes) surface receptors, stimulated superoxide production and PLDs activation in human monocytes, which promoted monocytes resistance to microbial infection [102]. Previous literature supports the idea that PLDs are associated with a variety of neutrophil responses, including actin polarization, migration, adhesion, and reactive oxygen species (ROS) production (Fig. 5). In vivo experiments demonstrated that PLD1 rather than PLD2 was able to regulate ROS production after stimulation by various factors in neutrophils [103]. PLD1 and PLD2 play regulatory roles in the polarization of macrophages. PLD1 activates LPS-MyD88 axis by coupling with Toll-like receptor 4 (TLR4) to drive the M2 transition of macrophages, whereas PLD2 activates JAK3 signal by coupling with interleukin-4 receptor to drive the transition of macrophages to M1 [104].

Adhesion is essential for the mobilization of innate immune-related cells to the site of infection and injury. A series of phosphate-dependent signal transduction pathways contribute to the regulation of adhesion function, including PKC family kinases downstream of the PLDs signaling pathway. PA produced by PLDs can be converted to DAG activating the PKC family [105], or PA can induce the activation of PI4-phosphate 5-kinase (PI4P5-K) to stimulate PI [4, 5] P2 synthesis [106]. PLD1 knockdown significantly inhibited the adhesion function of phagocytes, accompanied by a reduction in total cellular F-actin (Fig. 5) [107]. PLD1 activation is involved in regulating the initial phase of neutrophils and macrophages adhesion, regulating the cytoskeleton-dependent antimicrobial response of myeloid leukocytes [97]. Conversely, chemokine receptors can regulate PLDs activity, and the roles of different receptors are different, which needs to be further explored. The modification of phospholipase D activity and isoform expression is widely involved in the development, maturation, and activation of bone marrow cells, including the proliferation, secretion of myeloblasts, and the activation of NADPH oxidase [108]. Sex differences in proinflammatory leukotrienes in human monocytes may be caused by PLD activity. In monocytes, androgen activates ERK, leading to reduced PLD activity (apparently attributed to the PLD1 isoform) and DAG levels, which are required for full induction of 5-lipoxygenase (5-LO, a key enzyme in leukotriene synthesis) activity [109].

#### Non-classical PLDs: emerging players in immune cell dynamics

PLD3 and PLD4, non-classical phospholipase D, participate in immune regulation dependent on their exonuclease activity. As 5’ ssDNA and ssRNA exonucleases, PLD3 and PLD4 are essential for regulating TLRs and cytoplasmic nucleic acid sensing pathways. Recognition of endocytic nucleic acids is a key component of the innate immune response. TLR is a key protein family that recognizes endocytic nucleic acids and plays a key role in the immune system. As a member of the pattern recognition receptors (PRRs) family, it serves as primary sentinels of innate immunity, detecting pathogen and damage associated molecular patterns (PAMPs/DAMPs) through conserved structural domains. TLR7 and TLR8, located in the endosomal/lysosomal fraction, can recognize ssRNA, and TLR9, also located in the endosomal/lysosomal fraction, is able to recognize DNA containing CpG [110]. Short ssDNA is also a substrate of PLD3 and PLD4, and PLD3/4 can regulate TLR9 downstream signaling by inhibiting the binding site when they compete for the substrate [9]. In the previous study, TLR9 was explained to exert immunosuppressive regulation across granulocytic-lymphoid compartments through MyD88-dependent inhibitory feedback loops [111], and the negative regulatory Mechanism: Activates NF-κB signalingmechanism of PLD3 and PLD4 on TLR9 signaling suggested the regulatory role of PLD3 and PLD4 in immunity. In the absence of PLD3 and PLD4, DC became more sensitive to TLR13 ligand and TLR7 ligand recognition, proving that PLD3 and PLD4 function as lysosomal nucleic acid scavengers, enzymatically degrading immunogenic RNA/DNA species to maintain immune tolerance, avoiding inflammatory and immune responses [112]. The expression range of PLD4 is relatively narrow compared to PLD3, and it is highly expressed in plasmacytoid dendritic cells and B cells in peripheral blood mononuclear cells samples, not expressed on the surface of other lymphocytes [48]. *Pld4* mutation can cause changes in the immunophenotype of mice [113], and it can promote the activation of M1 macrophages [114]. These results suggest that PLD4 plays a more significant role in immune regulation than PLD3, and the specific mechanism needs to be further elucidated **(**Fig. 5**)**.

### PLDs dysregulation in autoimmune pathogenesis

PLDs play regulatory roles in many immune-related diseases, mainly autoimmune diseases. Autoimmune diseases, caused by the immune system’s immune response to the self-components, originates from breakdown of self-tolerance mechanisms, wherein dysregulated adaptive immunity erroneously targets endogenous epitopes through autoreactive T/B cell activation. *Pld1* expression is increased in mouse models of autoimmune diseases, including experimental autoimmune encephalomyelitis (EAE) and experimental autoimmune myocarditis by participating in the activation of macrophages in the lesion [115, 116]. The incidence of various immune-mediated central nervous system (CNS) diseases, such as EAE and multiple sclerosis (MS), is significantly lower in *Pld1*^*−/−*^ mice than in WT mice, which may be mediated by lymphocyte adhesion and migration, suggesting that *Pld1* may be a therapeutic target for the progression of CNS [117]. In addition, PLD1 can promote the development of rheumatoid arthritis (RA), and the use of PLD1 inhibitors can improve disease progression by regulating pathogenic T cells and B cells [118]. At the cellular level, PLD1 knockdown reduced the metastasis and inflammatory response of RA-fibroblast-like synoviocytes by inhibiting the activity of NF-κB and Wnt/β-catenin pathways [119]. PLD1 expression can also regulate the function of Treg cells and participate in the pathogenesis and progression of SLE through competitive binding with miRNAs or trans-regulation mechanism [120]. PLD2 can regulate lymphocytes, neutrophils, and macrophages. The role of PLD2 in systemic inflammatory response syndrome or sepsis has been reported in the literature. *Pld2* KO mice showed increased survival and reduced vital organ damage in experimentally induced sepsis, and these alterations may be due to increased bactericidal activity, promoted lymphocyte apoptosis, and reduced systemic inflammation caused by *Pld2* deletion [121]. Down-regulation of PLD2 in peripheral T lymphocytes is positively correlated with the development of oral lichen planus, a common T lymphocyte-mediated autoimmune disease [122]. *Pld1*- or *Pld2*-deficient mice have impaired phagocytosis of macrophages and suppressed neutrophil recruitment, which may affect the body’s immune and inflammatory responses [123]. PLDs have complex regulatory effects on immune cells, and these regulatory effects also affect the body’s anti-infection ability. PLDs activity has been reported to promote rapid endocytosis of influenza viruses, allowing the virus to escape detection by innate immunity **(**Fig. 5**)** [124].

When *Pld3* and *Pld4* were both deficient, mice died within 2–3 weeks of birth due to severe liver inflammation, the liver had extensive CD68^+^ cell infiltration, immunometabolism disorder, and significant elevation of related inflammatory factors. However, *Pld4* deficiency did not cause mouse death, but exhibited a TLR9-driven inflammatory syndrome, splenomegaly, upregulation of MHC II, and alterations in the number of monocytes, platelets, NK, and B1 cells, similar to macrophage activation syndrome in humans [9]. The similar functions of PLD3 and PLD4 contribute to their complementary effects in vivo, which can ameliorate the immune disorders caused by the loss of one or the other. As a susceptibility gene for SLE, mutations in *Pld4* can affect SLE disease progression by promoting SLE-consistent autoimmune phenotypes [113]. These data suggest that PLD4 can be a potential therapeutic target for immune diseases and that PLD3 can act synergistically with PLD4. PLD6 is involved in the infiltration of immune cells in patients with ulcerative colitis [125], and the exact mechanism has not been elucidated (Fig. 5).

## PLDs in tumor immunity: mechanisms of immune regulation and therapeutic targeting

### PLDs expression in tumors

PLDs are key regulators of cancer pathogenesis, influencing cellular processes including proliferation, invasion, metastasis, immune evasion, metabolic reprogramming, and therapy resistance. PLDs exhibit distinct expression patterns across different tumor types, and their functional roles in malignancies are dynamically regulated by variations in their expression levels and catalytic activity.

#### PLDs expression and tumor cell proliferation

**Fig. 6:**
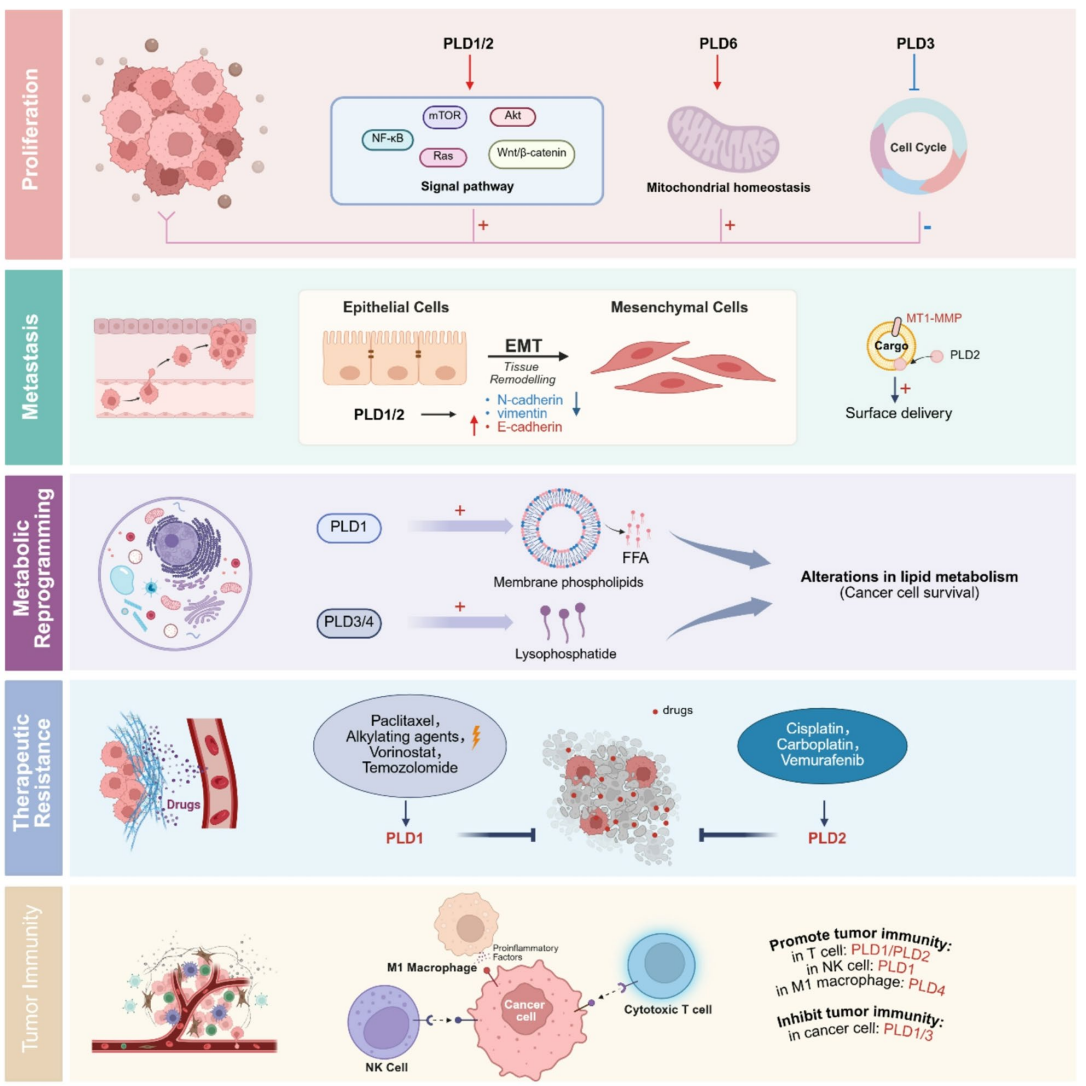
PLDs dysregulated in cancer progression. PLDs dysregulation impacts multiple hallmarks of cancer. Proliferation: PLD1 and PLD2 promote proliferation by activating various oncogenic signaling pathways (e.g., mTOR, Akt, NF-κB). PLD6 facilitates proliferation by hydrolyzing mitochondrial membrane phospholipids to maintain mitochondrial homeostasis. In contrast, PLD3 acts as a tumor suppressor in breast cancer by inducing cell cycle arrest. Metastasis: PLD1 and PLD2 primarily enhance the epithelial-mesenchymal transition (EMT) program. Additionally, PLD2 promotes metastasis by regulating the extracellular delivery of MT1-MMP. Metabolic Reprogramming: PLDs contribute to lipid metabolic rewiring, primarily by generating free fatty acids. PLD3 and PLD4 modulate lysophosphatidic acid levels, influencing downstream tumor metabolic pathways. Therapy Resistance: Upregulation of PLD1 and PLD2 expression is implicated in resistance to various chemotherapeutic agents. Immunomodulation: Context-dependent roles are observed, elevated PLDs expression in immune-activated cells (e.g., CD8^+^ T cells, NK cells, M1 macrophages) may promote anti-tumor immunity, whereas increased PLDs expression in tumor cells can suppress it

It has been identified that compared with normal gastric tissues, the expression of PLD1 protein was upregulated in the gastric tumor tissues [126]. The PLD1-specific inhibitor could significantly inhibit the mTOR signaling pathway to regulate the cell cycle of gastric cancer cells and lead to suppressed gastric cancer cell proliferation [127]. Transcriptomic analysis of clinical specimens revealed a significant upregulation of PLD1 in nasopharyngeal carcinoma tissues compared to non-cancerous tissues. Mechanistically, PLD1 drives tumor proliferation by activating the NF-κB signaling pathway and its downstream target genes [44]. Under the interaction of Cofilin 1, PLD1 activates the protein kinase B (Akt) signaling pathways, thereby promoting the growth of hepatocellular carcinoma cells [128]. PLD1 mediates the crosstalk among multiple major signaling pathways including PI3K/Akt, Wnt/β-catenin, and retinoblastoma 1/E2 factor signaling pathway, thereby promoting the survival and malignancy of colorectal cancer cells [129]. In normal and benign prostatic hyperplasia tissues, PLD1 expression is localized to basal cells and certain stromal cells. In prostate cancer, however, PLD1 expression shifts predominantly to luminal cells. Targeting with a novel specific PLD1 inhibitor has been demonstrated to reduce survival in prostate cancer [130]. PLD1 may promote the progression of cervical cancer by regulating the phosphorylation of ERK and activating the Ras signaling pathway [45]. In glioma cell lines, overexpression of PLD1 can promote their proliferation and migration capabilities [131] (Table 1; Fig. 6).

**Table 1:** Role and mechanisms of PLDs in cancer hallmarks

Cancer Hallmark	PLD Isoform	Expression & Impact	Core Mechanisms & Associated Cancers
Proliferation	PLD1	Up → Promotes	Mechanism: Regulates mTOR signaling Cancer: GC ^(127)^
	PLD1	Up → Promotes	Mechanism: Activates NF-κB signaling Cancer: NPC ^(44)^
	PLD1	Up → Promotes	Mechanism: Activates Akt signaling Cancer: HCC ^(128)^
	PLD1	Up → Promotes	Mechanism: Mediates the crossover between multiple major signaling pathways Cancer: CRC ^(129)^
	PLD1	Up → Promotes	Mechanism: Unknown Cancer: PC ^(130)^
	PLD1	Up → Promotes	Mechanism: Activates Ras pathway Cancer: CC ^(45)^
	PLD1	Up → Promotes	Mechanism: Unknown Cancer: Glioma ^(131)^
	PLD2	Up → Promotes	Mechanism: Unknown Cancer: SCC ^(132)^
	PLD3	Down → Inhibits	Mechanism: Induction of mitotic arrest Cancer: BRCA ^(134)^
	PLD3	Down → Inhibits	Mechanism: Unknown Cancer: OS ^(135)^
	PLD3	Up → Promotes	Mechanism: Unknown Cancer: GBM ^(136)^
	PLD5	Up → Promotes	Mechanism: Unknown Cancer: PCa ^(39)^
	PLD6	Up → Promotes	Mechanism: Activates Wnt/β-catenin pathway Cancer: CRC ^(138)^
	PLD6	Up → Promotes	Mechanism: Regulates mitochondrial function Cancer: BRCA ^(139)^
Invasion & Metastasis	PLD1	Up → Promotes	Mechanism: Activates NF-κB signaling pathway Cancer: NPC ^(44)^
	PLD1	Up → Promotes	Mechanism: Regulates mTOR signaling pathway Cancer: GC ^(126)^
	PLD1	Up → Promotes	Mechanism: Activates EMT process Cancer: HCC ^(128)^
	PLD1	Up → Promotes	Mechanism: Activates EMT process Cancer: CC ^(45)^
	PLD2	Up → Promotes	Mechanism: Promotes the transport of MT1-MMP Cancer: BRCA ^(50)^
	PLD2	Down → Inhibits	Mechanism: Inhibits EMT process Cancer: CRC ^(141)^
	PLD2	Up → Promotes	Mechanism: Activates EMT process Cancer: HGSC ^(142)^
	PLD5	Up → Promotes	Mechanism: Unknown Cancer: PC ^(143)^
Metabolic reprogramming	PLD1	Up → Promotes	Mechanism: Unknown Cancer: LC ^(144)^
	PLD3	Down → Inhibits	Mechanism: Alter the metabolism of lysophospholipids Cancer: PDAC ^(146)^
	PLD3	Up → Promotes	Mechanism: Unknown Cancer: Glioma ^(136)^
	PLD4	Up → Promotes	Mechanism: Unknown Cancer: CCRCC ^(147)^
Treatment resistance	PLD1	Up → Promotes	Mechanism: Metabolic reprogramming Cancer: LC ^(150)^
	PLD1	Up → Promotes	Mechanism: Unknown Cancer: Glioma ^(151)^
	PLD2	Up → Promotes	Mechanism: Regulates the rewiring of stemness genes Cancer: OC ^(153)^
	PLD2	Up → Promotes	Mechanism: Inhibits drug-induced DNA damage Cancer: Melanoma ^(154)^
Immune evasion	PLD1	Up (in CD8^+^T cell) → Inhibits	Mechanism: Activates TCR signaling ^(156)^
	PLD2	Up (in CD8^+^T cell) → Inhibits	Mechanism: Regulates the infiltration of CD8^+^ T cells Cancer: Melanoma ^(157)^
	PLD3	Up → Promotes	Mechanism: Reduces sensitivity to CD8^+^ T cells killing Cancer: Melanoma ^(158)^
	PLD1	Up → Promotes	Mechanism: Inhibits the phagocytic function of macrophages Cancer: CRC ^(161)^
	PLD2	/	Mechanism: unknown Cancer: BC ^(162)^
	PLD1	Up (in NK cell) → Inhibits	Mechanism: enhances the killing ability of NK cells Cancer: HL ^(163)^
	PLD4	Up (in M1 type macrophages) → Inhibits	Mechanism: Promote the secretion of pro-inflammatory factors Cancer: Colon cancer ^(114)^

*Abbreviations: GC* Gastric cancer, *NPC* Nasopharyngeal carcinoma, *HCC* Hepatocellular carcinoma, *CRC* Colorectal cancer, *PC* Prostate cancer, *CC* Cervical cancer, *SCC* Squamous cell carcinoma, *BRCA* Breast cancer, *OS* Osteosarcoma, *GBM* Glioblastoma, *HGCS* High-grade serous carcinoma, *LC* Lung cancer, *PDAC* Pancreatic ductal adenocarcinoma, *CCRCC* Clear cell renal cell carcinoma, *OC* Ovarian cancer, *HL* Hodgkin lymphoma

The expression level of PLD2 in squamous cell carcinoma tissue samples is significantly increased, and it has been found that the absence of PLD2 inhibited proliferation and promoted cell apoptosis in vitro [132]. Based on in vivo studies, PLD2 overexpression enhances stemness properties and tumorigenic potential, correlating with Wnt signaling pathway activation. Additionally, secreted PLD2 from cancer cells induces senescence in neighboring fibroblasts, suggesting its role in modulating the tumor microenvironment [133]. At present, little is known about non-classical PLD members in tumors. In breast cancer, PLD3 expression is downregulated compared to normal adjacent tissues. Functionally, PLD3 exerts its tumor-suppressive effect by binding to cyclin-dependent kinase 1 (CDK1) via p53-ZEB1 axis, inhibiting its expression, inducing mitotic arrest, and ultimately suppressing cell proliferation [134]. In the prognostic survival analysis of osteosarcoma, patients exhibiting elevated PLD3 expression levels demonstrated statistically superior survival outcomes compared to counterparts with diminished expression [135]. In addition, PLD3 is also associated with glioblastoma recurrence [136]. PLD5 promotes the proliferation, migration, invasion, and metastasis of prostate cancer cells [39], though its molecular mechanisms remain uncharacterized. PLD6, as a key enzyme regulating mitochondrial dynamics [137], enhances mitochondrial metabolism through its catalytic activity, activating the Wnt/β-catenin signaling pathway and promoting the occurrence of colorectal cancer [138]. PLD6 also affects YAP/TAZ function in mammary epithelial cells and breast cancer by regulating MYC-driven mitochondrial fusion and fission [139], while broader tumorigenic roles require further validation (Table 1; Fig. 6).

In summary, PLDs promote tumor cell proliferation through diverse mechanisms. PLD1 is frequently upregulated and drives cancer cell growth by activating key pathways including mTOR, NF-κB, Akt, and Wnt/β-catenin in various cancers. PLD2 enhances stemness and tumorigenesis, usually via Wnt signaling pathway and modulates tumor microenvironment. In contrast, PLD3 acts as a tumor suppressor in breast cancer. The roles of PLD5 and PLD6 are under elucidating, with PLD6 linked to Wnt signaling and mitochondrial metabolism. Critically, preclinical studies show that specific PLD1 inhibitors can reduce cancer cell viability, highlighting their significant therapeutic potential for cancer (Fig. 6).

#### PLDs expression in tumor migration and invasion

It was reported that the PLD1-specific inhibitor could inhibit the migration and invasion of gastric cancer [126]. In addition, after knockdown of PLD1, the migration ability of nasopharyngeal cancer cells significantly decreased, and the expression of migration (N-cadherin, vimentin) markers also decreased [44]. Similarly, in hepatocellular carcinoma, overexpression of PLD1 can increase the expression of N-cadherin and vimentin [128]. In human cervical cancer cell line CASKI, the knockout of PLD1 improved the expression of E-cadherin in tumor tissues, inhibited the expression of vimentin, and suppressed the EMT process [45]. PLD1 may promote tumor metastasis by directly interacting with ADAM10 (a disintegrin and metalloproteinase domain-containing protein 10) [140]. This interaction stabilizes the membrane localization of N-cadherin, thereby effectively inhibiting the shedding of its ectodomain (Table 1; Fig. 6).

Based on subtype specificity, PLD2 mainly regulates tumor migration and invasion. PLD2 plays a critical role in promoting breast cancer invasion and metastasis through multiple interconnected mechanisms. PLD2 drives breast cancer metastasis by regulating MMP-1 type matrix metalloproteinase (MT1-MMP, a key protease involved in local invasion and metastasis) surface delivery through a PA-KIF5B (the heavy chain of kinesin-1) dependent mechanism, facilitating invadopodia formation and cell invasion without affecting primary tumor growth [50]. In colorectal cancer tissues, PLD2 expression is significantly downregulated compared to adjacent normal tissues. In vitro experiments have demonstrated that the deficiency of PLD2 promotes cell invasion and metastasis in colorectal cancer by inducing EMT [141]. The expression of PLD2 is significantly higher in high-grade serous carcinomas (HGSC, the most common and clinically aggressive ovarian cancer tissue type with extremely high drug resistance) than in ovarian cancer and solid metastatic tumors, suggesting a new potential role of PLD2 in mediating the EMT of this cancer type [142]. PLD5 has recently been reported to be associated with metastasis in prostate cancer [143], though its specific mechanisms remain unelucidated. Furthermore, the roles of other non-canonical PLDs in tumor metastasis have not yet been extensively investigated, representing an area requiring further exploration (Table 1; Fig. 6).

#### PLDs expression and metabolic reprogramming

Aldehyde dehydrogenase A and PLD1 jointly regulate the production of PA, influencing the generation of downstream metabolites, thereby causing the metabolic reprogramming of lung cancer cells treated with alkylating agents or radiation [144]. The mechanism of this process still requires further exploration. Under glucose deprivation conditions, PLD1 can regulate membrane phospholipids to produce free fatty acids to maintain cancer cell survival [145] (Table 1; Fig. 6).

The tumor suppressor protein p53 serves as the molecular core of cellular metabolism. Through high-throughput non-targeted lipidomics analysis of pancreatic ductal adenocarcinoma (PDAC) cells, the absence of p53 leads to a decrease in PLD3 expression level, thereby altering the metabolism of lysophospholipids [146]. PLD3 expression shows prognostic significance in PDAC patients, highlighting its role in tumor metabolic reprogramming through lipid metabolism regulation. Spatial transcriptomics reveal PLD3 as a key lipid metabolism gene upregulated in tumor-associated macrophages of recurrent glioblastoma. Its expression correlates with patient survival across multiple cohorts, suggesting a role in metabolic reprogramming of the tumor microenvironment [136]. These findings position PLD3 as a potential mediator of immunosuppressive metabolism in GBM progression. Reprogramming of lipid metabolism is a hallmark of the progression of clear cell renal cell carcinoma (CCRCC). Through transcriptome analysis of clinical samples, lipid metabolism-related phospholipase PLD4 affects the prognosis of CCRCC [147]. However, the specific mechanisms of lipid metabolism targeting have not yet been clarified (Table 1; Fig. 6).

PLDs demonstrate significant potential in tumor metabolic reprogramming through their roles in lipid metabolism. PLD1 and PLD2 contribute to cancer cell survival under stress conditions by generating PA and regulating fatty acid metabolism [6], while PLD3 emerges as a key lipid metabolic regulator in pancreatic cancer and glioblastoma microenvironment, showing clear prognostic significance. However, the role of PLD5 and PLD6 in metabolic reprogramming remains deserted. These findings collectively position PLD family members as promising therapeutic targets for disrupting metabolic adaptations in various cancers, though their specific mechanisms require further elucidation.

#### PLDs expression and treatment resistance

Paclitaxel is one of the most widely used chemotherapeutic agents for treating solid cancers such as breast, gastric, and cervical cancers. It directly targets mitochondria by inducing reactive oxygen species (ROS) production, leading to cell death [148]. In metastatic cancer cells, however, PLD1 drives the accumulation of lipid droplets (LDs), which protect cells from paclitaxel-induced stress [149]. Specifically, PLD1-mediated LD formation helps reduce ROS levels and maintain endoplasmic reticulum (ER) homeostasis during treatment, thereby enhancing resistance to paclitaxel. These findings identified PLD1 as a promising therapeutic target for overcoming chemoresistance in metastatic cancer. It is common that resistance to alkylating agents and radiation therapy in lung cancer. Recent studies have implicated metabolic reprogramming as a potential determinant of sensitivity and resistance to these anticancer therapies [150]. Following treatment with alkylating agents or radiation, lung cancer cells can upregulate the expression of PLD1. It has been demonstrated that PLD1 confers enhanced resistance to both alkylating agents and radiation in lung cancer cells, with the underlying mechanism potentially involving PLD enzymatic activity-driven metabolic reprogramming of tumor cells [144]. Vorinostat is a histone deacetylase (HDAC) inhibitor considered as an effective anticancer agent. However, when used against glioblastoma, it transcriptionally upregulates PLD1 expression via PKCζ-Sp1 axis, thereby inducing resistance to itself [151]. Furthermore, PLD1 can also induce the resistance of glioblastoma to the chemotherapy compound temozolomide [152]. The precise mechanism by which PLD1 mediates resistance to anticancer therapies in glioblastoma has not been fully elucidated, representing an important direction for future studies (Table 1; Fig. 6).

Hypoxia in solid tumors is also a key contributor to chemotherapy resistance. It induces the upregulation of PLD2, which subsequently promotes resistance to cisplatin and carboplatin in ovarian cancer models both in vitro and in vivo by rewiring the expression of stemness-associated genes [153]. PLD2 drives vemurafenib resistance in BRAF V600E-mutant melanoma through a forkhead box protein A1 (FOXA1)/PLD2 regulatory axis [154]. Mechanistically, PLD2 upregulation protects melanoma cells from drug-induced DNA damage, while its suppression significantly enhances vemurafenib sensitivity. The synergistic effect observed with PLD inhibitors and vemurafenib highlights PLD2 as a promising therapeutic target to overcome drugs resistance.

PLDs, particularly PLD1 and PLD2, emerge as pivotal regulators of metabolic reprogramming in tumor chemoresistance. They promote resistance to diverse agents, including paclitaxel, alkylators, radiation, HDAC inhibitors, and targeted therapies through mechanisms such as lipid droplet formation, ROS reduction, stemness regulation, and DNA damage protection. Their consistent role across cancer types positions PLDs as promising therapeutic targets for reversing cancer treatment resistance, while other PLDs’ role in treatment resistance remains further studied (Table 1; Fig. 6).

### PLDs in tumor immunity

The tumor microenvironment (TME), a highly structured ecosystem, is the environment surrounding tumors, including immune components (e.g., tumor-infiltrating lymphocytes, myeloid-derived suppressor cells), stromal elements (including cancer-associated fibroblasts, neovascular networks), and biochemical mediators. Immune cells have a dual role in tumors. Immune cells can play an immune surveillance role to prevent tumor progression when they adopt anti-tumor phenotypes. Immune cells, under the influence of TME, can adopt a pro-tumor phenotype that allows tumor escape or even supports TME, thereby promoting tumor progression [130]. Anti-tumor immunity, as an important mechanism for the body to resist the occurrence and development of tumors, refers to the ability of the body’s immune system to recognize and eliminate tumor cells.

#### PLDs regulate CD8^+^ T cells killing ability

In recent years, PLDs, as important cell signal transduction molecules, not only play roles in tumor cells, but also affect the function of immune cells in the tumor microenvironment. PLDs have different regulatory effects on tumor immune infiltration in different tumor types, which may increase anti-tumor immunity or inhibit its immune infiltration. T cells play a central role in cancer immunotherapy [155]. studies have shown that PLD1 plays an important role in TCR signaling, and its enhanced activity can promote the proliferation of T cells and the secretion of cytokines (such as interleukin-2), thereby regulating anti-tumor immune responses [156]. The growth of subcutaneously transplanted tumor cells (B16 melanoma and Lewis lung cancer cell lines) in *Pld2*-knockout mice was increased compared with wild type mice, and the mechanism was due to the decreased infiltration of CD8^+^ T cells caused by the loss of PLD2, leading to decreased antitumor immunity [157]. PLD2 is required for CD8^+^ T cells to inhibit tumor growth in melanoma. PLD2 deficiency can reduce the number of CD8^+^ T cells in the spleen of mice, possibly through the Ras/ERK pathway [157]. Studies have shown that antigens of lymphocytes can up-regulate the PLD signals of specific T lymphocytes, suggesting that PLD signals are mainly required for the proliferation of CD8^+^ T cells with killing ability and CD4/CD25 T cells with helper killing ability in mice, key lymphocytes involved in antitumor immunity [91]. Using CRISPR-Cas9 library to perturb the genome in human melanoma cells, PLD3 knockout sensitized Mel624 melanoma cells to T cell-mediated killing [158] (Table 1; Fig. 6).

#### PLDs regulate tumor-associated immune cells beyond CD8^+^ T cells

With tumor growth, the ability of anti-tumor immunity decreases, the number of immunosuppressive cells (including Tregs and regulatory B cells) and the infiltration of neutrophils and tumor-associated macrophages (TAMs) increase. PLDs play key roles in the signal transduction of macrophages and neutrophils, participate in leukocyte chemotaxis and cancer cell invasion, and may regulate the dynamic balance of their tumor immune microenvironment [159]. In breast cancer, PLD inhibitors treatment can inhibit the recruitment of tumor-associated neutrophils and static M2 macrophages to the tumor, and promote the activation of M1 macrophages, beneficial to anti-tumor immunity [160]. PLD1 inhibition enhances macrophage phagocytosis of colorectal cancer cells through surface expression of costimulatory molecules, and PLD1 inhibitor can increase its anti-tumor effect in combination with anti-PD-L1 antibody [161]. In *Pld2* KO mice, *Pld2* deficiency promotes bladder cancer progression through immunosuppression in the TME, mainly dependent on interleukin-1β (IL-1β) secreted by TAMs [162]. Activation of the ARF6-PLD1 axis enhances the killing effect of natural killer (NK) cells on malignant Hodgkin lymphoma (HL) through antibody-dependent cell-mediated cytotoxicity [163]. In recurrent GBM, PLD3 was upregulated in microglia/macrophages (Iba1^+^) and negatively correlated with patient survival, a finding implicating an important role of PLD3 in tumor immunity in GBM [136]. PLD4 knockdown can reduce the secretion of pro-inflammatory cytokines in M1 macrophages and rescue the apoptosis of cancer cells by co-culturing with colon cancer cells, suggesting that PLD4 is involved in the activation of M1 macrophages in tumors [114] (Table 1; Fig. 6).

PLD family plays a key role in promoting tumor growth and inhibiting anti-tumor immune response in most tumors. The specific mechanism has not been fully elucidated, and further research is needed to find it out.

### Tumor immunotherapy potential of PLDs

**Fig. 7:**
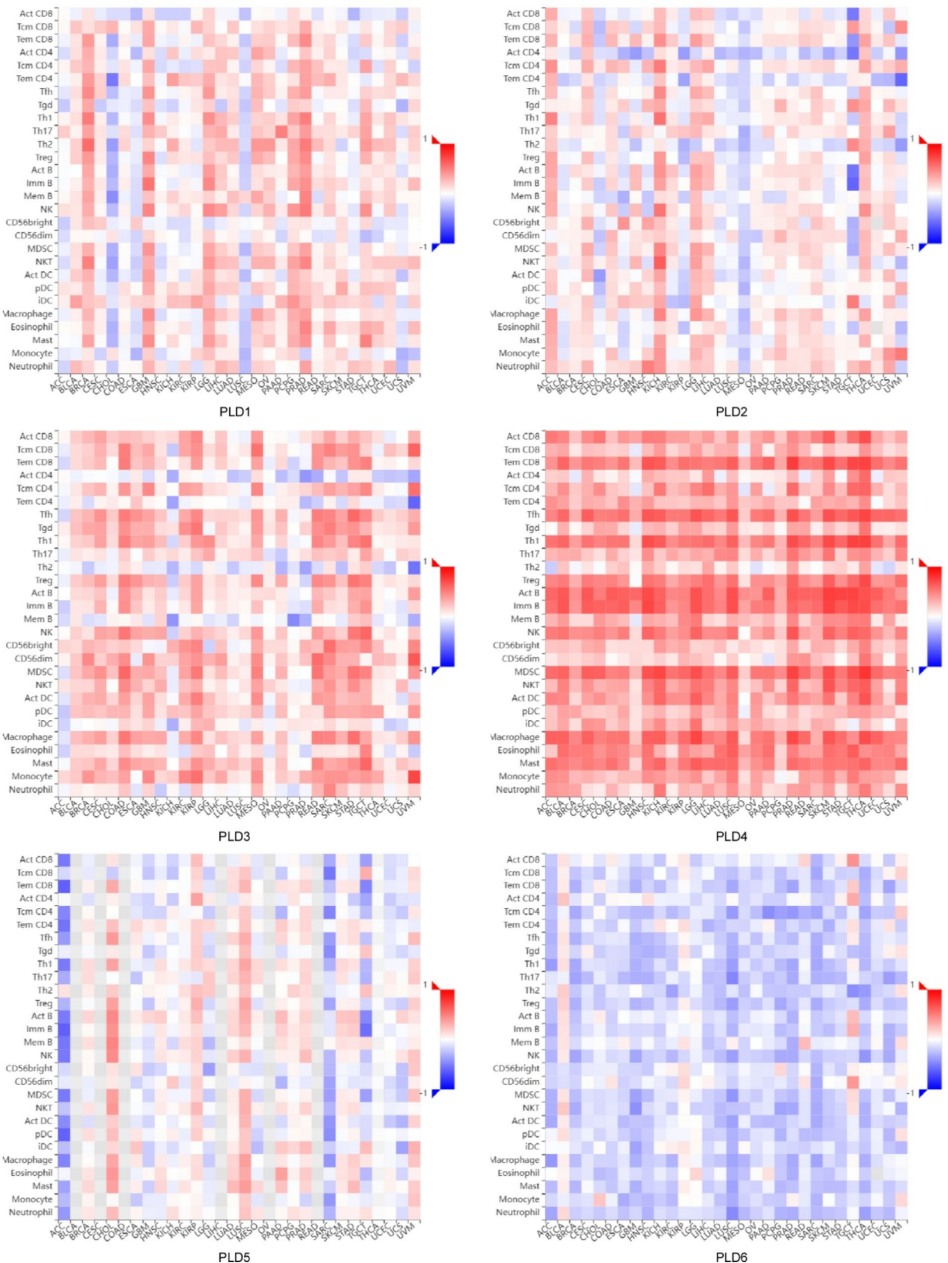
Spearman correlation heatmaps between expression of PLDs and TILs (Y axis) across human cancers (X axis). PLDs as pivotal modulators of tumor-immune crosstalk, orchestrating immunosuppressive microenvironment formation through multifaceted mechanisms. Through the TISIDB portal website, the relationship between the expression of PLD subtypes and the abundance of tumor infiltrating lymphocytes (TILs) was analyzed. Most tumors showed a correlation between PLD subtypes and TIL levels, with PLD3 and PLD4 exhibiting significant positive correlations, and PLD6 showing a significant negative correlation

Tumor infiltrating lymphocytes (TILs) are a kind of immune cell that exist in the tumor microenvironment, including cytotoxic T cells, T helper cells, regulatory T cells, B cells, etc. They can directly or indirectly participate in the immune response and affect tumor growth and treatment response. Elucidation of tumors and immune cell interactions will help predict immunotherapy responses and develop novel immunotherapy targets. The relationship between the expression of PLD family members and the abundance of TILs was analyzed through the TISIDB portal to further understand their important roles in the tumor immune microenvironment [164]. The expression of classical PLDs (PLD1, PLD2) and non-classical PLDs (PLD3, PLD4) in most tumors was positively correlated with TILs abundance, especially PLD4. The highly strong association between PLD4 and TILs may be due to its tissue-specific association (high expression with immune cells). However, PLD6 expression was inversely correlated with TILs abundance in most tumors. The differences of PLD family members also make them play different roles in tumor immunity (Fig. 7). These correlations suggest that PLDs are key regulators of immune response in the tumor microenvironment and have the potential to be applied in tumor immunotherapy. Understanding the exact mechanism and function of the PLDs will help to develop new therapeutic strategies aimed at enhancing anti-tumor immunity and provide new directions and strategies for further exploration of novel immunotherapy targets.

### Microenvironment-specific bottlenecks in targeting PLDs for cancer therapy

The PLD family generally promotes tumor growth and suppresses anti-tumor immunity across most cancers, though certain subtypes paradoxically enhance immune responses in specific contexts. Key unresolved mechanisms remain. The “double-edged sword” nature of classical PLDs (PLD1/2) in tumor immunity. Classical PLDs demonstrate a dual-function profile in tumor immunity within immune cells such as T cells, macrophages, and NK cells, PLD1 and PLD2 enhance anti-tumor immunity via enzymatic activity-mediated signaling pathways (e.g., TCR/mTOR/NF-κB/Ras pathway). Specifically, PLD1 facilitates TCR-induced T cell proliferation and IL-2 secretion, while PLD2 sustains CD8⁺ T cell infiltration and cytotoxic function. Furthermore, PLD inhibitors have been shown to reverse M2 macrophage polarization and augment NK cell-mediated cytotoxicity. In contrast, within tumor cells, these same molecules, particularly PLD1, promote oncogenic processes including proliferation, migration, and immune evasion through cancer-related signaling pathways. PLD2 contributes to the establishment of an immunosuppressive tumor microenvironment via the IL-1β-TAMs axis. This functional dichotomy may arise from differences in subcellular localization (e.g., membrane-bound oncogenic signals versus immune synapse activation), metabolic reprogramming within the tumor microenvironment. Lactate accumulation suppresses immunity while may activate tumor-associated PLD and context-dependent signaling networks [165]. Future therapeutic strategies should focus on achieving spatiotemporal specificity and combining PLD inhibition with immune checkpoint blockade to enable precise targeting.

In-depth exploration of the functions of non-classical PLDs as metabolic enzymes in tumor metabolism. PLD3, PLD4, and PLD6 possess dual enzymatic functions, encompassing phospholipase and nuclease activities, and exert complex, multi-faceted regulatory effects on the tumor microenvironment. While some functions of these isoforms have been identified, their underlying molecular mechanisms remain largely unexplored. Notably, PLD3 exhibits opposing roles in different cancers, acting as a tumor-suppressive factor in breast cancer yet functioning as a tumor-promoting agent in glioblastoma. Resolving this paradox necessitates comprehensive analysis from two perspectives, cell-type-specific interaction profiles (e.g., nuclear localization in breast cancer vs. potential lysosomal localization in glioblastoma) and metabolic reprogramming (e.g., regulation of lipid metabolism). The distinct roles of PLD4 in colorectal cancer and renal cell carcinoma highlight its significance in shaping the tumor immune microenvironment. PLD4 may primarily promote anti-tumor immunity through its nuclease activity, influencing macrophage polarization and serving as a regulator of endosome-derived nucleic acid signaling. By degrading immunostimulatory nucleic acids (ssDNA/ssRNA), it inhibits the Toll-like receptor 9 signaling pathway, preventing excessive inflammation and supporting the M1 phenotype. Additionally, its phospholipase activity may play a secondary role in lipid signal transduction. In clear cell renal cell carcinoma, PLD4 co-expresses with lipid metabolism-related genes and correlates with patient prognosis. It remains to be determined whether PLD4 regulates prostaglandin synthesis or cholesterol metabolism via its phospholipase activity, thereby modulating tumor-associated macrophage function. PLD6, localized to mitochondria, holds a central position in tumor metabolism and metastasis, warranting further exploration. Current breast cancer models must be expanded to better understand its role. PLD5, implicated in prostate cancer progression, remains poorly characterized at the mechanistic level, representing a promising area for future research. Of particular interest is the potential involvement of non-classical PLD3/4/6 in metabolic crosstalk between tumor cells and immune cells, potentially mediated by lipid mediator release or degradation of tumor-derived nucleic acids, ultimately shaping an immune-activating or inhibitory microenvironment.

## PLDs inhibitors

**Fig. 8:**
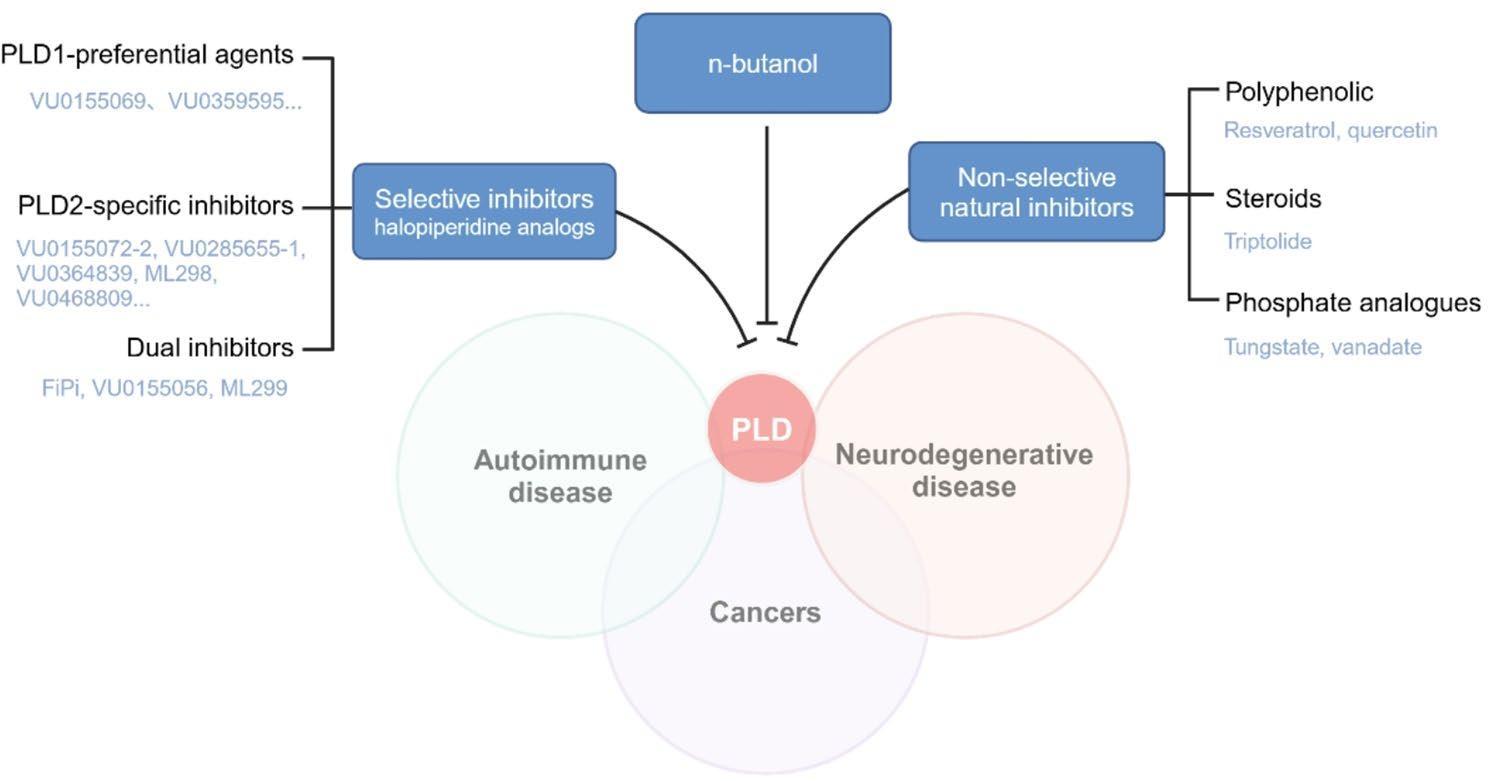
PLDs inhibitors. PLDs serve as pathophysiological modulators in multiple disease axis, including neurodegenerative disorders, autoimmune disease, and cancers. Targeting PLDs with various inhibitors may provide promising strategies for human diseases. Classification of PLDs inhibitors: primitive non-selective agents and isoform-targeted inhibitors

The expanding understanding of PLDs’ pathophysiological roles in diverse disease contexts, particularly its implications in neurodegeneration, autoimmunity, and oncogenesis has catalyzed growing enthusiasm within the scientific community for developing targeted PLDs inhibitors. In the early stages, small molecule inhibitors capable of modulating PLDs enzyme function were identified. However, most of these inhibitors were non-selective natural products. These include indirectly regulated polyphenolic natural products such as resveratrol [166, 167] and quercetin [168], steroids such as triptolide [167], and directly regulated phosphate analogues such as tungstate and vanadate [169]. Additionally, n-butanol can serve as an inexpensive inhibitor of PLDs but is limited by its high concentration requirements and lack of specificity (Fig. 8) [170].

In 2007, halopiperidine and related analogs were discovered to act as PLD2 inhibitors [171]. These analogs include the now widely used 5-fluoro-2-indolyl deschloropiperidine (FiPi) derivatives. Further experimental evaluations revealed that these compounds could inhibit both PLD1 and PLD2, functioning as dual PLD1 and PLD2 inhibitors. Halospermine, as an atypical antipsychotic drug [172], exhibits limitations when utilized as a PLDs inhibitor. To overcome the non-specific nature of natural products and the constraints of halopenamine-based inhibitors, researchers initiated the development of isoenzyme-selective PLDs inhibitors derived from halopenamine, nonselective PLDs inhibitors (e.g., FiPi, VU0155056, and ML299) [173]. By introducing or modifying various functional groups, the study identified that the addition of (S)-methyl groups significantly enhanced PLD1 selectivity, exemplified by highly potent PLD1 inhibitors such as VU0155069 and VU0359595 [174]. Simultaneously, researchers further optimized PLD2 selectivity and identified highly effective PLD2 inhibitors (e.g., VU0155072-2, VU0285655-1, VU0364839, ML298, and VU0468809) (Fig. 8) [6].

Currently, PLDs inhibitors are being employed in tumor therapy. Dual PLD1/2 inhibitors FiPi and 5W0, as well as the PLD1-specific inhibitor EVJ, have been shown to significantly reduce the proliferation of human prostate cancer cells [130]. The combination of PLDs inhibitors with radiotherapy enhances the cytotoxic effects on cancer cells, while the specific inhibitor FiPi blocks EGF-induced calcium signal transduction in human breast cancer cells [144, 175, 176]. PLDs inhibitors also hold promise in tumor immunotherapy. PLD1 inhibitors decrease the expression of “do not eat me” signals, increase “eat me” signals, and enhance macrophage phagocytosis of colon cancer cells [161]. Furthermore, PLDs inhibitors regulate oncogenic pathways and immune escape mechanisms in lung cancer cells [177]. FiPi reduces glial scar formation and promotes motor function recovery after spinal cord injury by inhibiting PLDs activity. PLDs inhibitors demonstrate potential therapeutic efficacy in rheumatoid arthritis, systemic lupus erythematosus, and other diseases through the modulation of T-cell activation and inflammatory cytokine release.

The development of PLD isoform-specific inhibitors is crucial for elucidating the distinct pathological mechanisms mediated by individual PLDs and for advancing our understanding of disease initiation and progression. Currently, the most highly selective PLD inhibitors target PLD1 and PLD2. For instance, the indole derivative 3r selectively inhibits PLD1 by binding to its F614 and F789 residues, with no observed activity against PLD2. This inhibitor exerts anti-tumor effects through a dual mechanism involving signal inhibition and immune activation [177]. Highly selective drugs need to target specific structural sites of the subtypes in order to distinguish between PLD1 and PLD2 and exert their therapeutic effects with precision. Furthermore, the functional duality of PLD1 and PLD2 in tumor cells versus immune cells complicates the development of targeted therapies. Research on inhibitors targeting non-classical PLD isoforms remains limited and faces unique challenges. The similarity in structure between PLD3 and PLD4 increases the difficulty in developing specific inhibitors. Tissue-specific functional paradox, such as the inhibitory effect of PLD3 on tumor growth in breast cancer cells and its promoting effect on tumor growth in glioma cells, which necessitates the development of delivery systems capable of achieving sub-organellar specificity. And the challenge of regulating dual enzymatic activities, phospholipase, and nuclease within the same protein.

## Perspective

The subcellular localization, functional roles, and enzymatic activities of PLD isoforms are systematically reviewed in this review. Canonical PLDs (PLD1/2) mediate signal transduction cascades through catalytic generation of PA or protein-protein interactions, thereby regulating cellular physiology. While PLD3 and PLD4 share structural and functional similarities with partial compensatory effects, their isoform-specific roles arise from distinct tissue distributions, PLD3 predominates in the central nervous system, whereas PLD4 is enriched in immune-related tissues. PLD6, localized to mitochondria and testes, modulates mitochondrial membrane dynamics via CL hydrolysis. Functional diversity among PLD family members (PLD1-6) stems from evolutionary divergence in catalytic domains and substrate preferences. Canonical PLD1/2 rely on the HKD motif-mediated phosphodiesterase activity to generate PA, activating downstream signaling pathways. In contrast, non-canonical PLD3/4 exhibit atypical acyltransferase activity regulating lysosomal lipid metabolism and possess ssDNA/RNA exonuclease activity, enabling their participation in lysosomal homeostasis and immunoinflammatory balance. PLD6 (mitoPLD) coordinates mitochondrial dynamics through cardiolipin hydrolysis. These enzymatic distinctions provide structural foundations for targeted drug design, PLD1-selective inhibitors like VU0155069 mimic the HKD domain transition state, while PLD3/4 inhibitor development may focus on their unique transmembrane domains. The development of specific inhibitors should focus on the specific functions and organ-specific tissue distribution of PLDs in diseases, moving beyond the traditional enzyme activity inhibition paradigm and towards function-specific regulation. PLDs critically regulate immune cell functionality (T cells, B cells, neutrophils, macrophages) via associated signaling pathways, offering therapeutic prospects for immune-related diseases. PLD1/2 further orchestrate multidimensional roles in the tumor immune microenvironment (TIME) through lipid signaling networks, influencing tumor progression, immune evasion, and therapy resistance. The TISIDB portal reveals isoform-specific expression of PLD3/4 in tumor-infiltrating immune cells, suggesting their immunotherapeutic potential. However, mechanistic insights into their TIME regulation remain limited, particularly their context-dependent roles (e.g., PLD3/4-mediated TLR7/8/9 signaling modulation), necessitating further functional dissection. Spatial transcriptomics will be essential for mapping PLD-enriched immunometabolic niches within TME, resolving isoform-specific spatial distributions and their functional crosstalk with immune checkpoints, thereby illuminating context-dependent PLD roles in metabolic reprogramming and immune evasion. Elucidating PLD pathophysiology could unlock novel strategies to modulate their activity for disease management. While research on canonical PLDs (including inhibitor applications) is relatively advanced, non-canonical PLDs investigations remain nascent. While *Pld1* and *Pld2* knockout mouse models have been extensively employed in studies of metabolic disorders, autoimmune diseases, and cancer, and *Pld3/Pld4* knockouts have been utilized for immune-related research, the absence of *Pld5* and *Pld6* knockout models has created a critical knowledge gap in understanding their functional mechanisms. There is an urgent need to establish tissue-specific conditional knockout models for PLD5 and PLD6 to elucidate their disease-related mechanisms, thereby addressing fundamental gaps in PLD research. This review synthesizes the multifaceted roles of PLD family members in disease contexts, aiming to advance mechanistic understanding of non-canonical PLDs, expand clinical applications of canonical PLDs, and explore future directions for isoform-targeted therapies. The multifaceted regulatory properties of PLDs position them as hub targets bridging metabolic reprogramming and immune editing. However, realizing their therapeutic potential requires overcoming the tripartite challenges of subtype selectivity, tissue specificity, and toxicity control. Systematic decoding of spatiotemporal PLDs network dynamics will pioneer innovative combinatorial therapies for precision medicine.

## Data Availability

Not applicable.

## Abbreviations

PLD: Phospholipase D
PKC: Protein kinase C
mTOR: Mammalian target of rapamycin
PA: Phosphatidic acid
PX: Phox consensus sequence
PH: Pleckstrin homology
PIP2: Phosphatidylinositol 4,5-bisphosphate
PI(4, 5)P_2_: Phosphatidylinositol-(4,5)-bisphosphate
DAG: Diacylglycerol
AA: Amino acid
TM: Transmembrane
ER: Endoplasmic reticulum
TLRs: Toll-like receptors
BMP: Bis(monoacylglycero)phosphate
CL: Cardiolipin
MK: Megakaryocyte
LPA: Lysophosphatidic acid
PIP3: Phosphatidylinositol 3,4, 5-triphosphate
TCF: T cell factor
NF-κB: Nuclear factor kappa-B
MAPK: Mitogen-activated protein kinases
EMT: Epithelial-mesenchymal transition
RAS: Rat sarcoma
Nrf2: NF-E2-related Factor-2
SLE: Systemic lupus erythematosus
PC: Phosphatidylcholine
PE: Phosphatidylethanolamine
PG: Phosphatidyglycerol
PI: Phosphatidylinositol
PS: Phosphatidylserine
PAP: Phosphatidic acid phosphohydrolases
DGK: Diacylglycerol kinases
PD: Parkinson's disease
AD: Alzheimer's disease
ALS: Amyotrophic lateral sclerosis
IRS: Insulin receptor substrate
BCR: B cell surface receptors
TCR: T cell-antigen receptor
PI5K: Phosphatidylinositol 5-kinase
ROS: Reactive oxygen species
PI4P5-K: PI4-phosphate 5-kinase
5-LO: 5-lipoxygenase
PRRs: Pattern recognition receptors
PAMPs/DAMPs: Pathogen- and damage-associated molecular patterns
EAE: Experimental autoimmune encephalomyelitis
CNS: Central nervous system
MS: Multiple sclerosis
RA: Rheumatoid arthritis
Akt: Protein kinase B
ADAM10: A disintegrin and metalloproteinase domain-containing protein 10
MT1-MMP: Matrix metalloproteinase-1 type matrix metalloproteinase
LD: Lipid droplet
KIF5B: Kinesin-1 heavy chain
TME: Tumor mircroenvironment
TAMs: Tumor-associated macrophages
IL-1β: Interleukin-1β
TILs: Tumor infiltrating lymphocytes
FiPi: 5-Fluoro-2-indolyl deschloropiperidine
GC: Gastric cancer
NPC: Nasopharyngeal carcinoma
HCC: Hepatocellular carcinoma
CRC: Colorectal cancer
PC: Prostate cancer
CC: Cervical cancer
SCC: Squamous cell carcinoma
BRCA: Breast cancer
OS: Osteosarcoma
GBM: Glioblastoma
HGSC: High-grade serous carcinoma
LC: Lung cancer
PDAC: Pancreatic ductal adenocarcinoma
CCRCC: Clear cell renal cell carcinoma
OC: Ovarian cancer
HL: Hodgkin lymphoma
